# Circadian rhythm disruption by PARP inhibitors correlates with treatment toxicity in patients with ovarian cancer and is a predictor of side effects

**DOI:** 10.1016/j.ebiom.2025.105764

**Published:** 2025-05-16

**Authors:** Deeksha Malhan, Janina Hesse, Nina Nelson, Kay Stankov, Jessica Nguyen, Ouda Aboumanify, Josefin Garmshausen, Gunther Rogmans, Bastian Czogalla, Jens Gerber, Martin Koch, Tomáš Kupec, Oliver Tomé, Ralf Witteler, Mustafa Deryal, Michael Eichbaum, Jalid Sehouli, Elena Ioana Braicu, Angela Relógio

**Affiliations:** a Institute for Systems Medicine and Faculty of Human Medicine, MSH Medical School Hamburg, Hamburg, Germany; b Leibniz-Institute for Resilience Research (LIR), Mainz, Germany; c Johannes Gutenberg University Medical Center Mainz, Mainz, Germany; d Institute for Quantitative and Computational Biosciences (IQCB), Johannes-Gutenberg University, Mainz, Germany; e Stat4med (Ainovate GmbH), Frankfurt, Germany; f North-Eastern German Society of Gynecological Oncology (NOGGO e.V.), Berlin, Germany; g Institute for Theoretical Biology (ITB), Charité—Universitätsmedizin Berlin, Corporate Member of Freie Universität Berlin, Humboldt-Universität zu Berlin, and Berlin Institute of Health, Berlin, Germany; h ZAGO- Zentrum für ambulante gynäkologische Onkologie, Krefeld, Germany; i Department of Obstetrics and Gynecology, LMU University Hospital, LMU Munich, Munich, Germany; j Städtisches Klinikum Dessau, Frauenheilkunde und Geburtshilfe, Dessau, Germany; k Department of Gynecology and Obstetrics, Hospital Anregiomed Ansbach, Ansbach, Germany; l Department of Obstetrics and Gynecology, University Hospital Aachen, Aachen, Germany; m ViDia Christliche Kliniken Karlsruhe, Department of Gynecology and Obstetrics, Karlsruhe, Germany; n Universitätsklinikum Münster, Klinik für Frauenheilkunde und Geburtshilfe, Münster, Germany; o Center for Gynecology, Caritas Klinikum St. Theresia-Saarbruecken, Saarbruecken, Germany; p Helios Dr. Horst Schmidt Kliniken Wiesbaden, Department of Gynecology and Obstetrics, Wiesbaden, Germany; q Department of Gynecology with Center for Oncological Surgery, Charité - Universitätsmedizin Berlin, Campus Virchow Klinikum, Berlin, Germany; r Tumorbank Ovarian Cancer Network, Charité - Universitätsmedizin Berlin, Corporate Member of Freie Universität Berlin, Humboldt-Universität zu Berlin, and Berlin Institute of Health, Berlin, Germany; s TimeTeller GmbH, Hamburg, Germany

**Keywords:** Circadian rhythms, Ovarian cancer, Circadian profiles, Chronotherapy, Adverse events, Mathematical modelling, PARP inhibitors, Rucaparib

## Abstract

**Background:**

Ovarian cancer is among the most lethal malignancies in women. The advent of PARP inhibitors (PARPi) has improved outcomes. However, treatment-related toxicity remains a critical challenge, impacting patient quality of life and treatment adherence.

**Methods:**

In a circadian sub-study of the MAMOC trial—a double-blind, phase III study—42 patients (FIGO stage IIIA-IV) were randomised in a 2:1 ratio to receive rucaparib or placebo. In a subset of these patients, we performed differential gene expression and rhythmicity analysis on up to 800 genes, including clock and clock-controlled genes. Machine learning algorithms and mathematical modelling were employed to simulate patient-specific toxicity profiles and to explore correlations between gene expression patterns and treatment-related side effects.

**Findings:**

Our analysis revealed significant disruptions in circadian rhythms, specifically in the expression of the core clock genes *BMAL1* and *PER2*, following treatment. These disruptions strongly correlated with the severity and frequency of side effects, including nausea and fatigue, displaying opposite trends between the placebo and rucaparib-treated groups. K-means clustering successfully distinguished rucaparib-treated patients from those receiving placebo based on *BMAL1* phase and gene expression profiles. In addition, rucaparib therapy also altered the expression of several clock-controlled genes, including *SIRT1*, *BRCA1*, *BRCA2*, and *TP53*. Notably, our data suggest that individual differences in circadian rhythms may lead to distinct 24-h toxicity profiles among patients.

**Interpretation:**

These findings suggest that circadian rhythm dysregulation may contribute to the toxicity of PARPi therapy. Aligning treatment timing with circadian rhythms could mitigate these adverse effects, and improve patient outcomes.

**Funding:**

This study was funded by the Dr. Rolf Schwiete Stiftung and the MSH Medical School Hamburg, Germany. The MAMOC trial (ClinicalTrials.gov: NCT04227522) was funded by 10.13039/100014487Clovis Oncology, United States.

**Research in context**

**Evidence before this study**

Patients with high-grade ovarian cancer often face a poor prognosis despite advancements in platinum-based and non-platinum-based therapies. Developing novel therapeutic strategies tailored to disease stage and molecular characteristics is critical for improving patient outcomes and quality of life. Targeted therapies, such as bevacizumab and PARP inhibitors (PARPi), have significantly improved progression-free survival (PFS) in patients with high-grade ovarian cancer. While previous studies have evaluated the use of bevacizumab alone and PARPi, particularly in patients with *BRCA1/2* mutations. Limited data exist on how patients without *BRCA1/2* mutations respond to PARPi following bevacizumab therapy.

Additionally, PARP1 is known to interact with the core circadian clock network, which regulates several cancer hallmark genes. However, the potential influence of circadian clock alterations on the efficacy of PARPi has not been investigated. We conducted a PubMed search for relevant studies published up to October 08, 2024, using the search terms ((“PARP inhibitor” [All Fields] OR “rucaparib” [All Fields] OR “olaparib” [All Fields] OR “niraparib” [All Fields]) AND “maintenance therapy” [All Fields] AND (“ovarian cancer” [All Fields]) AND (“BRCA” [All Fields] OR “BRCA1” [All Fields] OR “BRCA2” [All Fields] OR “BRCA1/2” [All Fields])), without language restrictions. Twelve published phase III trials were identified that assessed PARPi in the treatment of ovarian cancer. These trials demonstrated that PARPi (olaparib, niraparib, rucaparib) significantly improved PFS in patients with high-grade ovarian cancer, both with and without *BRCA1/2* mutations, compared to placebo. Two phase III trials investigated the combination of PARPi with bevacizumab or chemotherapy. In the PAOLA-1 trial, olaparib with bevacizumab showed improved PFS compared to bevacizumab alone, while in NRG-GY004, olaparib, either alone or with cediranib, did not surpass chemotherapy in efficacy. However, no studies have specifically examined the use of PARPi maintenance therapy following bevacizumab maintenance in patients without *BRCA1/2* mutations. Furthermore, none of the identified studies investigated the effect of PARPi on the core circadian clock network, a potential factor that could influence therapeutic outcomes.

**Added value of this study**

This circadian sub-study of a randomised, double-blind, placebo-controlled phase III trial offers two key contributions. First, it investigates the sequential and dual use of targeted maintenance therapies—bevacizumab and rucaparib—in patients without *BRCA1/2* mutations. Second, in this sub-study, we specifically examine circadian clock-associated molecular changes and their correlation with treatment-related adverse events. Importantly, we identified significant circadian disruptions in patients undergoing rucaparib therapy, which were closely linked to the severity and frequency of side effects. This provides insights into the interaction between circadian rhythms and cancer therapy, suggesting that optimising treatment timing based on individual circadian profiles could help reduce toxicity and improve patient outcomes.

**Implications of all the available evidence**

Ovarian cancer remains one of the deadliest malignancies in women, with limited treatment options for advanced stages. While PARPi have significantly improved outcomes, especially in *BRCA*-mutated patients, treatment-related toxicity remains a major challenge. Our study reveals that rucaparib therapy disrupts circadian rhythms, particularly in the expression of core-clock genes *BMAL1* and *PER2*, which correlates with the severity and frequency of side effects. These findings suggest that circadian dysregulation may contribute to treatment toxicity. Incorporating chronotherapy—tailoring treatment timing to align with patients' circadian rhythms—could reduce adverse effects, improve treatment adherence, and potentially enhance the efficacy of PARPi in patients with ovarian cancer. This approach may pave the way for more personalised and effective cancer treatments.

## Introduction

Ovarian cancer remains one of the most lethal cancers affecting women worldwide, accounting for 3.7% of cases and 4.7% of cancer deaths in the year 2020, primarily due to late-stage diagnosis.^1^ Despite advancements in screening methods, the majority of ovarian cancer cases are diagnosed at an advanced stage, where the prognosis is often poor. The current standard of care, which involves cytoreductive surgery followed by platinum-based chemotherapy, has shown limited success in extending survival rates.^2^ Recently, the advent of targeted therapies, particularly poly (ADP-ribose) polymerase (PARP) inhibitors, has brought new hope to patients with ovarian cancer.^3^ PARP inhibitors (PARPi), such as rucaparib, target DNA repair pathways and have demonstrated efficacy, particularly in patients with *BRCA1/2* mutations.

Recent phase III trials, including OReO,^4^ PRIME,^5^ SOLO1,^6^ PAOLA-1,^7^ SOLO2,^8^ NRG-GY004,^9^ NORA,^10^ NOVA,^11^ PRIMA,^12^ ARIEL3,^13^ ATHENA-MONO,^14^ and ARIEL4,^15^ have explored the efficacy and safety of PARPi as maintenance therapy for ovarian cancer, especially in patients with *BRCA* mutations and homologous recombination deficiency (HRD).^4^, ^5^, ^6^, ^7^, ^8^, ^9^, ^10^, ^11^, ^12^, ^13^, ^14^, ^15^, ^16^ SOLO1 utilised olaparib as a standard maintenance therapy in newly diagnosed *BRCA*-mutated advanced ovarian cancer, significantly improving progression-free survival (PFS). PAOLA-1 demonstrated that combining olaparib with bevacizumab notably improved PFS in HRD-positive patients, regardless of *BRCA* status. SOLO2 further confirmed olaparib's effectiveness in recurrent *BRCA*-mutated ovarian cancer. PRIMA validated niraparib's benefit in newly diagnosed ovarian cancer, particularly in HRD-positive cases. NORA corroborated niraparib's efficacy in Chinese patients with recurrent ovarian cancer, aligning with results from NOVA. ARIEL3 showed that rucaparib offered significant PFS benefits across various subgroups, including *BRCA*-mutated and HRD-positive patients. ATHENA-MONO highlighted rucaparib's effectiveness as a single-agent maintenance therapy in newly diagnosed ovarian cancer, while ARIEL4 supported rucaparib's use in heavily pre-treated *BRCA*-mutated patients. Collectively, these trials affirm PARPi as a vital component of maintenance therapy in ovarian cancer, particularly for patients with *BRCA* mutations and HRD. These phase III trials collectively affirm the role of PARPi—such as olaparib, niraparib, and rucaparib—as effective maintenance therapies in both newly diagnosed and recurrent ovarian cancer, particularly among patients with *BRCA* mutations and HRD-positive tumours. The findings have led to the incorporation of PARPi into standard care, offering significant PFS benefits and improved patient outcomes across various clinical settings.

Despite these advances, there remains a critical need to optimise therapeutic strategies to reduce toxicity and improve outcomes. One promising approach that has gained attention in recent years is chronotherapy—aligning treatment with the patient's circadian clock.^17^ Administering chemotherapy at times when cancer cells are more vulnerable and healthy cells are less sensitive can reduce toxicity. For instance, drugs like irinotecan, 5-fluorouracil (5-FU), and oxaliplatin in colorectal cancer show better efficacy and tolerability when timed with circadian cycles.^18^

The circadian clock, an internal biological mechanism that operates on a 24-h cycle, plays a crucial role in regulating various physiological processes, including metabolism, immune response, and cell proliferation.^19^ At the molecular level, the circadian clock is formed by core-clock genes, which form transcriptional-translational feedback loops (TTFL) that maintain 24-h/circadian rhythmicity. The TTFL involves core-clock genes and proteins. The CLOCK and BMAL1 proteins form a heterodimer that activates the transcription of target genes, including *PER* (Period) and *CRY* (Cryptochrome), which accumulate and inhibit the activity of the CLOCK-BMAL1 complex, thereby repressing their own transcription.^20^ Additionally, the nuclear receptors REV-ERB and ROR regulate the expression of *BMAL1*, with REV-ERB repressing and ROR activating its transcription, contributing to the approximate 24-h oscillatory rhythm of the circadian clock. Disruption of circadian rhythms has been implicated in numerous diseases, including metabolic disorders,^21^ neurodegenerative diseases,^22^ and cancer.^23^ In cancer, dysregulation of the circadian clock has been linked to tumour development, progression, and response to therapy. Specifically, studies have shown that the circadian clock regulates DNA damage response pathways, and its dysregulation may contribute to genomic instability, a hallmark of cancer.^19^ For instance, in lung cancer, dysregulation of circadian genes like *BMAL1* and *PER2* has been associated with tumour growth and metastasis.^24^ In colorectal cancer, studies suggest that altered expression of circadian genes can influence the efficacy of chemotherapy, highlighting the potential for chronotherapy to optimise treatment timing.^25^ Similarly, in hepatocellular carcinoma, circadian disruption has been linked to increased susceptibility to DNA damage, suggesting that integrating circadian biology into treatment strategies could reduce tumour progression.^26^ These findings underscore the broader relevance of circadian rhythms across various cancer types and highlight the potential for chronotherapy to improve patient outcomes. Therefore, understanding the circadian clock's role in ovarian cancer, particularly in the context of PARP inhibition, could provide valuable insights into optimising therapeutic strategies.

Recent evidence has highlighted a direct interaction between PARP1, a key enzyme in DNA repair, and the CLOCK-BMAL1 complex, a central component of the circadian clock network. This interaction suggests that circadian rhythms may influence the efficacy of PARPi and that aligning PARPi therapy with the patient's circadian rhythm may enhance treatment outcomes. In addition to DNA repair, circadian rhythms also regulate other cellular processes relevant to cancer therapy, including cell cycle progression and apoptosis. Therefore, circadian dysregulation in patients with ovarian cancer undergoing PARP inhibition may contribute to variations in treatment response and toxicity. While PARPi have shown significant promise in treating ovarian cancer, there is growing interest in integrating chronotherapy—scheduling treatment in alignment with the body's circadian rhythms—into oncological practices. Chronotherapy, which involves timing drug administration to align with the body's natural rhythms, has shown promise in other cancer types and can be applied to improving outcomes in patients with ovarian cancer, and may offer a personalised approach to cancer care^17^ by enhancing treatment efficacy and reducing toxicity.^27^

This study aims to investigate circadian clock-associated alterations in patients with high-grade ovarian cancer undergoing maintenance therapy with rucaparib vs. placebo following platinum-based chemotherapy and bevacizumab maintenance therapy as part of the MAMOC clinical trial, and how circadian rhythms disruptions may inform chronotherapy strategies in ovarian cancer treatment. Specifically, we measured the circadian rhythmicity of core-clock gene expression in these patients before, during, and after rucaparib therapy and assessed how these rhythms are affected by rucaparib therapy. By characterising the circadian clock's role in ovarian cancer treatment, we aim to explore the potential mechanisms through which chronotherapy may contribute to reduced toxicity and improved quality of life in patients undergoing this therapy. The circadian rhythms analysis showed significant differences in the expression of core clock genes (*BMAL1* and *PER2*) between rucaparib-treated and untreated (placebo) patients. Rucaparib caused severe side effects. Our findings reveal that rucaparib therapy significantly disrupts circadian properties of *BMAL1* and *PER2*, with these alterations correlating with increased toxicity, including fatigue, insomnia, and nausea. Our results indicated a significant correlation between impaired circadian rhythms and the frequency and severity of side effects in these patients. The analysis of circadian profiles showed that disruption of the circadian rhythms in *PER2* correlates most strongly with the number of side effects, while the dysregulation of circadian rhythms in *BMAL1* correlates most strongly with the severity of side effects. Furthermore, gene expression analysis suggests that rucaparib-induced circadian disruptions extend beyond core-clock genes, affecting key cancer-associated genes such as *VEGFA*, *BRCA1*, *BRCA2*, *SIRT1*, and *TP53*. This study highlights the intricate relationship between circadian rhythm alterations and treatment toxicity, emphasising the potential role of chronotherapy in reducing side effects and improving patient outcomes.

By integrating circadian biology with current therapeutic strategies, this study seeks to advance our understanding of how circadian rhythms influence PARPi therapy in patients with ovarian cancer, and to explore the potential benefits of chronotherapy for patients with ovarian cancer. Ultimately, the goal is to optimise treatment timing to improve clinical outcomes while minimising toxicity, paving the way for more personalised and effective cancer therapies. The findings from this study could have broader implications for the role of circadian rhythms in cancer therapy and contribute to the growing field of chronotherapy in oncology.

## Methods

### Study design and patients

MAMOC is a randomised, double-blind, placebo-controlled phase III trial to investigate the sequential and dual approach of targeted maintenance therapies with bevacizumab and rucaparib. The trial was conducted in 26 hospitals and cancer centres in Germany, and the trial was closed in December 2023. We included patients with histologically confirmed, advanced (FIGO stage IIIA, IIIB, IIIC, or IV of the 2014 FIGO^28^ classification) high-grade serous or high-grade endometrioid (based on local histopathological findings) ovarian cancer, fallopian tube cancer, primary peritoneal cancer and clear cell carcinoma of the ovary in first-line therapy. In compliance with GDPR (General Data Protection Regulation), we adhered to the principle of data minimisation and only collected information essential for the study objectives. Patient characteristics such as race and ethnicity were not collected as these variables were not relevant to the study endpoints. All the patients were recruited through different hospitals within Germany. Furthermore, data on patients’ age at recruitment along with various clinical characteristics (cancer type, FIGO staging, etc.) were recorded.

Eligible patients were women from the age of 18 years without a mutation in *BRCA1* or *BRCA2*, confirmed by a central NGS analysis; with previous maintenance treatment of bevacizumab (for 12–15 months total duration, without treatment interruption); completed first-line platinum-taxane chemotherapy and at least stable disease after treatment with bevacizumab before randomisation, confirmed by a radiological assessment; with an Eastern Cooperative Oncology Group (ECOG) performance status of 0 or 1. Patients who had previously received any other PARPi in first-line therapy or radiotherapy within 6 weeks prior to study treatment were excluded. Patients provided written informed consent before participating in the trial and the circadian rhythms sub-study.

This sub-study was conducted within the MAMOC trial using an intention-to-treat (ITT) approach to evaluate molecular changes in the circadian clock of patients with ovarian cancer receiving rucaparib vs. placebo. The ITT population in MAMOC includes all patients who were randomised in the trial, regardless of whether they received the study treatment, and serves as the primary population in the MAMOC trial for analysing patient characteristics and efficacy endpoints such as PFS and overall survival.

### Ethics

The trial was approved by national and local regulatory authorities and ethic committees (ethical approval reference number: 19/0237–IV E 13; BfArM (Federal Institute for Drugs and Medical Devices, Germany) reference number: 4043608), designed and conducted in accordance with the German Medicinal Products Act–AMG (Arzneimittelgesetz), the ICH Harmonised Tripartite Guidelines for Good Clinical Practice, with applicable local regulations (including European Directive 2001/20/EC) and with the ethical principles laid down in the Declaration of Helsinki from 1996. The study was conducted and reported in accordance with the Strengthening the Reporting of Observational Studies in Epidemiology (STROBE) guidelines.^29^ This sub-study, aimed at investigating the putative role of circadian rhythms disruption in the occurrence of side effects in the analysed cohort. The study was conducted as part of the main MAMOC trial and received approval. The MAMOC study was approved in early 2020, with site activation beginning in the summer of that year. Patient recruitment was impacted by the COVID-19 pandemic, with a decline observed during its initial phase, after which it gradually improved. The trial recruitment stopped in December 2022. The CONSORT-CONSERVE checklist^30^ was completed to document the impact of the COVID-19 pandemic on patient recruitment in the MAMOC trial and in this sub-study. The trial was registered at ClinicalTrials.gov (Identifier: NCT04227522).

### Inclusion and exclusion criteria

Patients were selected based on the following inclusion criteria:

1. Informed Consent: Written informed consent obtained from participants before any study procedures.
2. Age: ≥18 years.
3. Diagnosis: Advanced ovarian, fallopian tube, or primary peritoneal cancer (FIGO stage IIIA-IV) confirmed as serous, high-grade endometrioid, or clear cell carcinoma, currently in first-line therapy.
4. Archival Tumour Tissue: Available for central NGS analysis with confirmed *BRCA*-negative status.
5. Bevacizumab Treatment: Completion of 12–15 months of bevacizumab, irrespective of dosage.
6. Chemotherapy Response: Completed first-line platinum-taxane chemotherapy with at least stable disease after bevacizumab treatment.
7. Randomisation Timeline: Randomisation 3–9 weeks after the last bevacizumab dose, with all major toxicities resolved to CTCAE grade 1 or better (excluding alopecia and peripheral neuropathy).
8. Performance Status: ECOG score of 0 or 1.
9. Organ Function: Normal organ and bone marrow function, defined as follows:
  a) Haemoglobin ≥10.0 g/dL without transfusion within 14 days.
  b) Absolute neutrophil count (ANC) ≥ 1.5 × 10ˆ9/L.
  c) Platelet count ≥100 × 10ˆ9/L.
  d) Total bilirubin ≤1.5 × ULN (≤2 × ULN for Gilbert's syndrome).
  e) AST/ALT ≤3 × ULN (or ≤ 5 × ULN if liver metastases present).
  f) Serum creatinine ≤1.5 × ULN and creatinine clearance >30 mL/min.
  g) INR ≤1.5 and aPTT ≤1.5 × ULN (for patients not on anticoagulants).
10. Reproductive Status: Female patients must be postmenopausal or have confirmed non-childbearing status prior to treatment. Those of childbearing potential require a negative pregnancy test within 3 days before starting rucaparib and must use highly effective contraception during treatment and for 6 months afterwards.

Patients were excluded based on the following criteria:

1. Cancer Type: Non-epithelial ovarian, fallopian tube, or peritoneal tumours (e.g., germ cell tumours, borderline tumours, or mucinous carcinoma).
2. Medical History: History of myelodysplastic syndrome, acute myeloid leukaemia, allogeneic bone marrow transplant, CVA, TIA, SAH, haemorrhagic disorders, or significant cardiovascular disease.
3. Recent Treatments: Radiotherapy within 6 weeks, major surgery within 4 weeks, or use of PARPi in first-line therapy.
4. Concurrent Treatments: Concurrent chemotherapy, other anti-cancer or anti-neoplastic hormonal therapy, or radiotherapy (hormone replacement and steroidal antiemetics allowed).
5. Brain and CNS: Suspected or confirmed brain metastases, spinal cord compression, or untreated CNS disease.
6. Coagulopathy and Bleeding Risk: History or active bleeding disorder or significant coagulopathy.
7. Injury and Healing: Significant injury within 4 weeks, non-healing wounds, active ulcers, or bone fractures.
8. GI Conditions: Clinically relevant bowel obstruction or conditions affecting drug absorption.
9. General Health Concerns: Conditions that may contraindicate investigational drug use or increase complication risk.
10. Pregnancy and Lactation: Pregnant, lactating, or childbearing women not using highly effective contraception.
11. Concurrent Clinical Trials: Participation in another clinical study before randomisation.
12. Drug Sensitivity: Known hypersensitivity to rucaparib or its components.
13. Infectious Diseases: known active HIV/AIDS, or chronic hepatitis B or C.
14. Other Active Cancer: Presence of another active malignancy requiring treatment.
15. Dependence: Patients dependent on the sponsor, CRO, site, or investigator.
16. Incarceration or Institutionalisation: Court-ordered incarceration or involuntary institutionalisation.

### Randomisation and masking

Randomisation occurred between 12 and 18 months after the start of bevacizumab treatment, within a window of 3–9 weeks after the last dose, and only if central *BRCA* testing confirmed a negative result. Patients were randomised in a 2:1 ratio to receive either rucaparib or placebo using a central interactive web response system (IWRS, Almac Clinical Services WebEZ, North Carolina, USA). Block randomisation (block size of three) was used, with stratification by surgery time point (adjuvant vs. neoadjuvant), surgical outcome (tumour-free vs. not tumour-free), study site, and treatment response (complete response vs. partial response/stable disease). To maintain blinding and reduce bias, all individuals involved in the trial, including patients and investigators, were unaware of treatment allocation, except for the Independent Data Safety Monitoring Board and pharmacovigilance team, who could unblind in emergency situations. Allocation concealment was ensured by generating randomisation lists exclusively through WebEZ, with neither the sponsor nor the CRO having access.

### Molecular diagnostics and treatment administration

To analyse the *BRCA* mutation status, a molecular diagnostic was carried out during the screening period via NGS analysis by Foundation Medicine (FMI) in Penzberg, Germany. Only cancer-relevant parts of the genome were sequenced from pseudonymised tumour samples, using the Foundation One CDx kit for solid tumours. *BRCA* wildtype was classified as no alteration in *BRCA1* and *BRCA2*. Patients assigned to the rucaparib arm received oral rucaparib 600 mg twice daily in 28-day cycles and placebo was administered in the same way. Placebo tablets were formulated to be indistinguishable from the rucaparib tablets. Administration of other simultaneous chemotherapy drugs, any other anti-cancer therapy or anti-neoplastic hormonal therapy, or simultaneous radiotherapy during the trial treatment period was not permitted. The precise intake times of rucaparib or placebo tablets were not recorded. However, patients were instructed to take two tablets (rucaparib or placebo) per day at 12-h intervals, ensuring that the medication was administered in the morning and evening. Maintenance treatment was terminated if one of these criteria was met: progression or relapse of disease during treatment (confirmed by RECSIST1.1 assessment), unacceptable side effects, occurrence of other diseases which interfere with study participation (e.g., secondary malignant neoplasm) or other appropriate reasons. Treatment with rucaparib/placebo was completed after 104 weeks (approx. two years). Treatment interruption and dose modification were permitted for CTCAE grade 2 or higher haematologic toxicity and grade 3 or 4 non-haematologic toxicity or grade 2 toxicity not adequately controlled by concomitant medications and/or supportive care. Dose reduction in both arms was possible in decimation steps of 100 mg twice daily. Two dose reduction steps were allowed, and for a third reduction step to 300 mg twice daily, consultation with the sponsor was required. If the toxicity did not improve despite dose reduction steps to 300 mg twice daily or matching placebo, or if dosing was interrupted for more than 14 consecutive days due to toxicity, treatment was to be discontinued, unless otherwise agreed and documented between the investigator and the sponsor. Primary efficacy analysis was defined as the time from the date of randomisation to the first documented disease progression according to and assessed by RECIST v1.1 or death from any cause, whichever occurred first. For patients that could not perform CT scans, MRI scans were permitted. However, the same method used to detect lesions at baseline 7 were to be used to follow lesions throughout the trial. Tumour assessment was performed at screening; every 6 months during maintenance therapy with rucaparib/placebo until end of treatment; at safety follow up, if no progression occurred according to RECIST 1.1 and according to local standard in the follow-up period.

### Collection of **P**atient **R**eported **O**utcomes **(PRO)**

Patient Reported Outcomes data were collected using: 1) European Organisation for Research and Treatment of Cancer Quality of Life Questionnaire-Core 30 (EORTC QLQ-C30).^31^ The EORTC QLQ-C30 is a 30-item questionnaire that assesses the quality of life of cancer patients across multiple domains, including physical, emotional, and social functioning. Functional, symptom, and global health scales were derived from the EORTC QLQ-C30, 2) European Organisation for Research and Treatment of Cancer Quality of Life Questionnaire—Ovarian Cancer Module (EORTC QLQ-OV28).^32^ The EORTC QLQ-OV28 is a 28-item-based questionnaire, that assesses the quality of life of patients with ovarian cancer, focussing on symptoms, treatment side effects, and psychosocial impacts. Functional and symptom scales were evaluated using the EORTC-QLQ-OV28 questionnaire, 3) Fatigue Symptom Inventory (FSI).^33^ The FSI is a 14-item-based questionnaire, that assesses the severity, frequency, and daily impact (perceived interference) of fatigue in clinical and research settings, 4) Short-Form Health Survey (SF-12).^34^ The SF-12 is a 12-item-based questionnaire, that measures physical and mental-health-related quality of life. Physical Component Summary (PCS) and Mental Component Summary (MCS) scores were calculated using the SF-12, 5) Everyday Memory Test (EMT).^35^ The EMT is a 13-item cognitive assessment tool used to evaluate the memory function of cancer patients in daily life situations, measuring recall and recognition of routine events and tasks, and 6) PRO Version of the Common Terminology Criteria for Adverse Events (PRO-CTCAE).^36^ PRO-CTCAE is a validated questionnaire used to assess symptomatic adverse events directly from patients, capturing the frequency, severity, and interference of treatment-related symptoms. In this study, the PRO-CTCAE assessment focused on eight key symptoms: decreased appetite, nausea, vomiting, constipation, diarrhoea, abdominal pain, insomnia, and fatigue. Additionally, any other symptoms reported by the patient were also recorded.

PRO data was obtained at screening, on day 1 of a cycle in 12-week intervals during maintenance therapy, at the safety follow-up visit (28 days after the last dose of the study drug) and in 12-week intervals during the follow-up period. After safety follow-up, patients were monitored for safety, efficacy and quality of life in 12-week intervals or according to local standards (CT/MRI) for a maximum of 2 years. PRO data used in this study were collected at screening, C1D1 (Cycle 1 Day 1), C4D1, C7D1, C10D1, C13D1, C14D1, C16D1, C19D1, safety follow-up (SFU), and during the follow-up (FU) period.

Patients were monitored for all adverse events (AEs) or severe adverse events (SAEs) or adverse events of special interest (AESIs) during study participation and until 30 days after the last dose of rucaparib/placebo. During the screening period, only SAEs which are related to protocol-mandated assessments were reported. From 30 days after the last dose of administration, only treatment-related SAEs and all AESIs, irrespective of causality, were reported. AEs and laboratory abnormalities were graded according to the NCI CTCAE grading system (Version 5.0). Adverse events of special interest included myelodysplastic syndrome, acute myeloid leukaemia and pneumonitis and related events. A list of all concomitant medications taken during the course of therapy was collected. Additionally, ECOG (Eastern Cooperative Oncology Group) performance status was recorded to evaluate patients' functional status during therapy. The ECOG performance status is a standardised scale used to measure a cancer patient's level of functioning and ability to perform daily activities.^37^

### Saliva sample collection and processing

Samples were collected and further processed using a non-invasive, saliva-based, *in vitro* diagnostic (IVD) TimeTeller kit (TimeTeller GmbH, Hamburg, Germany) currently applied for research use only purposes^38^, ^39^, ^40^ (https://www.timeteller-health.com/), following the manufacturer's instructions, and used for profiling the circadian rhythms among the patients in the rucaparib and the placebo groups. Unstimulated saliva samples were collected at the following times: Time 0-before the therapy (8 saliva samples during two days); Time 1–4 weeks after therapy-start (8 saliva samples during two days); Time 2–12 weeks after therapy-start (8 saliva samples during two days); Time 3–6 months after therapy-start (8 saliva samples during two days); Time 4–1 year after therapy-start (8 saliva samples during two days); Time 5- end of treatment (8 saliva samples during two days). The 8 saliva samples were collected over two consecutive days (Day 1: ca. 9 h, ca. 13 h, ca. 17 h, ca. 21 h; Day 2: ca. 9 h, ca. 13 h, ca. 17 h, ca. 21 h). Following collection, the samples were stored at −20 °C at the different participating hospitals, as per the provider's instructions, and then shipped to TimeTeller GmbH for further analysis. The expression levels of core-clock genes (*BMAL1*, *PER2*) and *PARP1* (QuantiTect primers (Qiagen, Hilden, Germany) *HS_ARNTL_1_SG* (Gene Globe ID QT00065933), *HS_PER2_1_SG* (Gene Globe ID QT00011207), *HS_GAPDH_1_SG* (Gene Globe ID QT00079247) and *HS_PARP1_1_SG* (Gene Globe ID QT00032690)) were normalised to *GAPDH* (ΔCt) and then to the mean expression value of each gene (time-course analysis), as described previously.^40^ Relative quantification was conducted using the 2^−ΔΔCt^ method.^41^

### NanoString nCounter data collection and analysis

RNA isolated from the saliva samples of selected patients, using the TimeTeller kit as described above, was also used to analyse 800 genes using the NanoString nCounter (NanoString Technologies, Seattle, WA, USA) with the nCounter PanCancer Pathway Standard Panel and the ECCN (Extended Core Clock Network)^42^ Plus Panel, following the manufacturer's instructions, and quantified using the nCounter SPRINT Profiler. The full list of genes measured by NanoString is provided in Table S1. Raw data files (Reported Code Count) along with Reported Library File (RLF) were retrieved for further analysis. Quality control and data normalisation were carried out using nSolver. A three-step normalisation was carried out: 1) normalisation based on the arithmetic mean of the positive controls, 2) subtraction of the arithmetic mean of the negative controls, and 3) normalisation by the geometric mean of the housekeeping genes. The normalised data was batch-corrected, and log2-transformed data was then utilised for the downstream analysis.

### Activity tracker

Physical activity and rest patterns were monitored using the Garmin Vivofit 4 tracker (https://www.garmin.com/). Patients were instructed to wear the tracker continuously, including during showering and swimming, while maintaining proper hygiene. To prevent potential bias in activity levels, the tracker display was preset to hide daily step counts from patients. Additionally, patients did not download the Garmin app, and data synchronisation was performed exclusively on-site by the study team. For patients with activity trackers, the number of steps was reported for each day over a patient-specific interval. For the same patient, the mean number of steps over one calendar month was related to the PRO data collected within the same month, if PRO data was collected during this month of activity tracker data. For some patients, tracker data was collected over intervals that spanned multiple PRO data collection dates, these were used as independent samples in the analysis.

### Correlation analysis

The relative expression and circadian properties (acrophase, amplitude, MESOR) of *BMAL1*, *PER2*, and *PARP1*, along with PRO, were compiled. The data was not normally distributed; therefore, Spearman correlation analysis was performed using the cor() function in R to calculate correlation coefficients, and *p*-values were obtained using the cor.test() function. The results were visualised with a heatmap generated by the *pheatmap* R package. Additionally, Spearman correlation analysis was conducted on a set of core-clock genes measured in selected patients using the same approach. To calculate the 95% confidence intervals (CIs), bootstrapping was carried out. Permutation testing was also performed to assess significance.

### Rhythmicity analysis

Gene expression data were processed using a multivariate harmonic regression model, *E_gc_* = *μ_g_* + *a_g_ cos* (*ϕ_c_*) + *b_g_ sin* (*ϕ_c_*) + *ε*, where “c” represents the condition, “g” represents the gene, and “ε” denotes Gaussian noise, as used previously.^43^ The model fits a periodic function (using cosine and sine terms) to the gene expression data, accounting for a 24-h cycle (a fixed period of 24 h used in our analysis), and helped to infer key circadian parameters, including amplitude, MESOR (Midline Estimating Statistic of Rhythm), and acrophase. MESOR represents the mean expression level over the 24-h cycle, serving as the baseline around which oscillations occur. Amplitude represents the difference between the peak expression and the MESOR, indicating the strength of oscillation. Acrophase refers to the time point at which the peak expression occurs within the cycle, providing information on phase timing.

### Differential expression analysis

Log2 normalised values from nSolver were used for the differential expression analysis of each measured patient. Differential expression analysis between the two groups was carried out using limma (version 3.0.6) Bioconductor R package.^44^ To obtain significantly up- and down-regulated gene sets, a cut-off of nominal *p* < 0.05 and a log fold change (logFC) ≥ 0.2 was used.

### Differential rhythmicity analysis

Differential rhythmicity analysis was carried out using Limorhyde^45^ R package to detect the changes in circadian parameters (acrophase, amplitude) due to clock alterations in patients who received therapy/no therapy vs. their Baseline. After pairwise comparison and *p* < 0.05, genes that depicted phase shift (≥ 4 h) or amplitude change were obtained.

### Mathematical modelling

The circadian expression profiles of the core-clock genes *BMAL1* and *PER2* were used to personalise a mathematical model combining core-clock rhythmicity and drug pharmaco-kinetics and -dynamics by Hesse, Müller and Relógio.^46^ The model simulates a gene regulatory network (transcription-translation network) of the core clock and clock-controlled genes relevant for drug action. A subset of the simulated genes controls equations for drug pharmaco-kinetics and -dynamics, such as import or activation of the drug, and cell death in response to the activated drug. Simulations from the model are used here to demonstrate the prediction of personalised circadian toxicity profiles resulting from the RNA expression values measured for core clock genes of a given patient.

The original model was fitted to the apoptosis dynamics in response to a chemotherapeutic agent (Irinotecan), which, like rucaparib, also interferes with DNA repair mechanisms.^46^ For personalisation, we free the parameters related to *BMAL1* and *PER2* expression, i.e. their degradation rates (original parameters dy1 and dy5), their fold change parameters (parameters a and i), their maximal transcription rates (parameters V1max and V5max), the parameters scaling the impact of their transcription factors CLOCK/BMAL and PER/CRY (‘kt1’, ‘ki1’, ‘kt5’, ‘ki5’), as well as the parameters that rule the protein translation of these transcription factors (‘dx1’, ‘dx2’, ‘dx5’, ‘dx6’, ‘kiz9’, ‘kex1’, ‘kiz4’, ‘kex2’, ‘kiz6’, ‘kiz7’). The freed parameters are fitted to the saliva gene expression of *PER2* and *BMAL1* by an evolutionary algorithm analogue to that used previously.^47^ Further, to allow for an alignment of the variable time in the model to real time, we have assumed a patient wake-up time of 7:00 AM. The phase of the fitted *BMAL1* expression is used to shift the model-intrinsic circadian rhythms of protein degradation, parameter *ϕ*PROTEIN, and of the apoptosis rate, parameter *ϕ*apop, by the phase difference between the original model and the fitted model.

### Ordinary least squares regression analysis

To identify significant predictors and assess the relationship between molecular data and PRO, ordinary least squares (OLS) regression analysis was performed in R. The underlying assumptions of OLS regression—linearity, independence, homoscedasticity, normality of residuals, and absence of multicollinearity—were tested. Linearity and homoscedasticity were assessed using residual plots, the normality of residuals was evaluated with the Shapiro–Wilk test and Q–Q plots, and multicollinearity was checked using variance inflation factors (VIF). To address the assumption of normality in the residuals, we implemented bootstrapping following the OLS regression analysis. *BMAL1* and *PER2* expression values or circadian properties were used as independent variables, while PRO measures (adverse events and quality of life) served as the dependent variable. We performed non-parametric bootstrapping with 25,000 iterations to assess the stability and precision of our OLS regression estimates. Prior to settling on this iteration number, we tested various numbers of bootstrap resamples and found that 25,000 iterations provided stable and consistent results. This non-parametric resampling technique, which generates empirical distributions of the test statistics by repeatedly sampling from the data with replacement, allowed us to obtain empirical confidence intervals and *p*-values for regression coefficients that do not rely on the normality of residuals, providing a more robust and reliable estimate of statistical significance. The strength and significance of associations were evaluated based on regression coefficients (beta estimate) and bootstrapped *p*-values. Additionally, bootstrap *p*-values were calculated by comparing the proportion of bootstrap resamples that were either greater than or less than zero, yielding two-tailed *p*-values for each coefficient. This approach mitigates potential issues related to non-normality and small sample sizes, ensuring the robustness and validity of our findings. We summarised the results using the 2.5th and 97.5th percentiles of the bootstrapped distribution to construct the 95% confidence intervals. The strength and significance of associations were evaluated based on regression coefficients (beta estimate) and bootstrapped *p*-values. Model performance was assessed using adjusted R-squared values.

### K-means clustering

To identify an optimal number of clusters based on core-clock gene expression or circadian properties that could differentiate between the placebo and rucaparib groups, k-means clustering was performed using the factoextra R package. The dataset was standardised before clustering, and the optimal number of clusters was determined by evaluating indices such as the silhouette method. Clustering patterns were then analysed to identify subgroups of patients with similar gene expression profiles or circadian patterns.

### Statistics

The primary objective of this study was to characterise the circadian clock-associated changes in patients by measuring the mRNA expression levels of core-clock genes and to evaluate how these expression phenotypes respond to therapy at various stages of treatment. Secondary objectives included: 1) to analyse the impact of circadian rhythm disruption on PRO such as quality of life, AEs, and SAEs, and 2) to explore the role of core-clock genes and clock parameters (amplitude, acrophase, and MESOR) in predicting potential treatment outcomes using statistical, machine learning methods, and mathematical modelling. Descriptive statistics were used for all analyses, and based on the distribution of data via normality testing, statistical significance analysis was carried out. Hedges' *g* was calculated to assess the effect size between patients who received corticosteroids and those who did not, as it provides a more conservative estimate by using the sample standard deviation, unlike Cohen's *d*,^48^^,^^49^ which uses the population standard deviation. Wilcoxon rank-sum test was carried out to evaluate the significant differences between the placebo and rucaparib groups. Given the small sample size, Fisher's exact test was used to assess significant differences in categorical variables between the groups. To assess whether the distribution of the rucaparib and placebo groups in the MAMOC cohort is similar to that in this sub-study, we performed a Kolmogorov–Smirnov test. OLS with bootstrapping was carried out to assess the association between the circadian clock-associated data and PRO in the placebo and the rucaparib groups. For each dependent and independent variable tested, the beta coefficient (β) was used to understand the dynamics of the relationships between variables in the placebo and the rucaparib groups. Analyses were done using R (version 4.4.1), and the significance level was set at 0.05. Multiple testing using Benjamini-Hochberg (FDR) correction was carried out. In addition to significance testing, we focused on effect size measures to assess the clinical relevance of our findings. For regression models, we report beta coefficients with 95% confidence intervals (CIs), while correlation analyses include Spearman correlation coefficients with CIs. These measures provide a clearer interpretation of effect magnitude and clinical importance beyond *p*-values.^50^, ^51^, ^52^

The linear regression lines and statistics of activity tracker data vs. PRO items were calculated using the python package statsmodels, fitting statsmodels.api. OLS with standard parameters. Using linregress (x,y) from scipy.stats with standard parameters, beta coefficients were calculated as the slope from the fit of linregress (zscore(x), zscore (y)), with zscore () from scipy.stats.mstats. For determining the 95% confidence interval of the regression coefficient, we used a linear regression fit of the python package statsmodels.api, model OLS, and the function conf_int (alpha = 0.05).

Given the exploratory nature of this study, no formal sample size calculations were performed. Instead, our findings provide a basis for future prospective studies with adequate statistical power, informed by the observed effect sizes.

### Role of funders

The funders of the study had no role in study design, data collection, data analysis, data interpretation, or writing of the research article.

## Results

### Study design overview and descriptive analysis of clinical outcomes

**Fig. 1:**
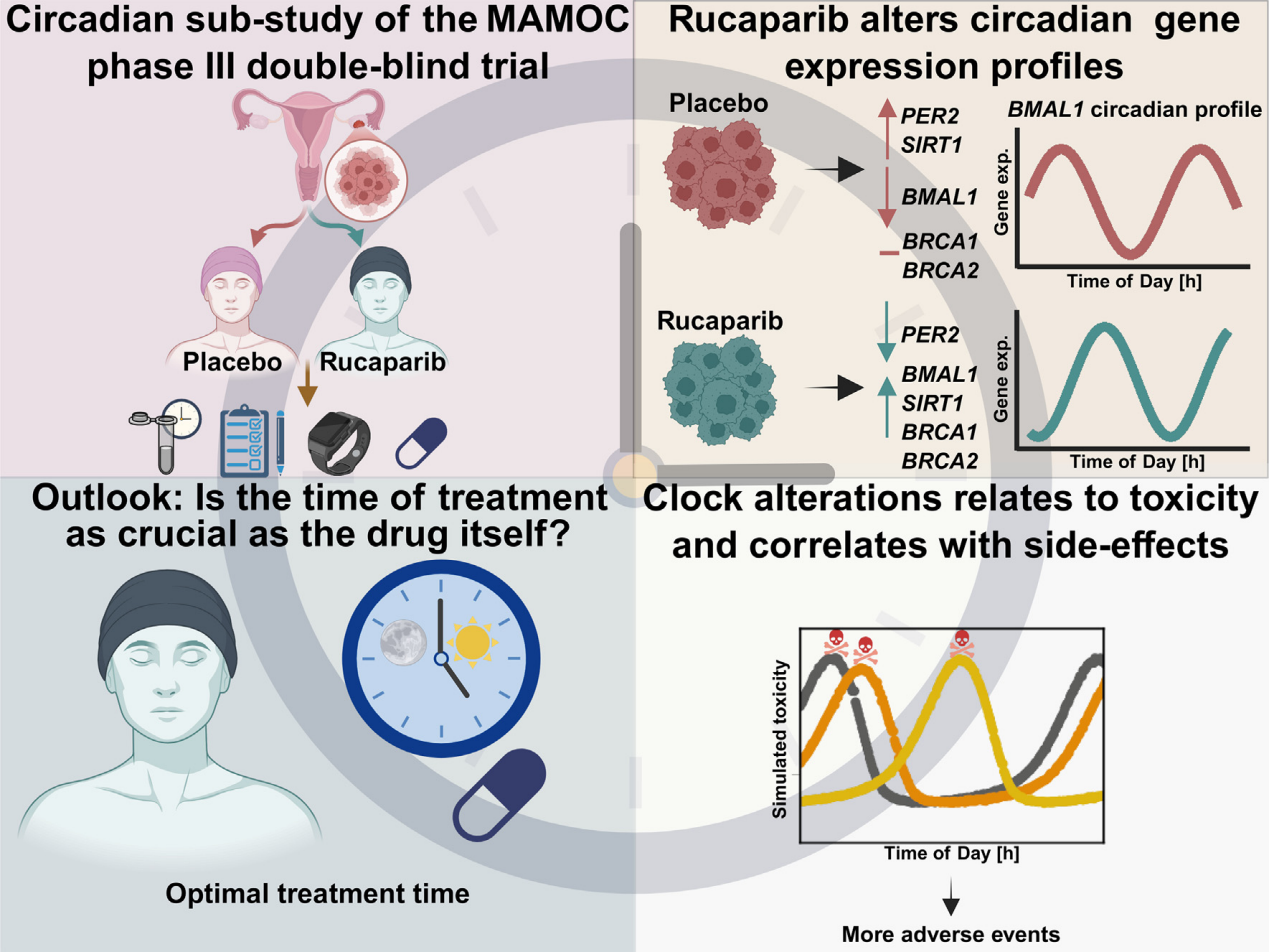
**Overview of the circadian sub-study within a Phase III double-blind, placebo-controlled trial.** A total of 42 patients with high-grade ovarian cancer were randomised to receive either rucaparib (N_P_ = 28) or placebo (N_P_ = 14). Saliva samples from 15 of those patients enabled molecular profiling of circadian rhythms during therapy. Gene expression analysis revealed treatment-specific circadian clock alterations in the rucaparib group, which correlated with side effects of the therapy. Mathematical modelling of toxicity profiles underscored the potential of circadian-based treatment timing to reduce side effects and improve outcomes (created in https://BioRender.com).

This work is a sub-study of the MAMOC trial (NOGGO-ov42: Rucaparib MAintenance After Bevacizumab Maintenance Following Carboplatin-Based First-Line Chemotherapy in Ovarian Cancer Patients; NCT04227522^53^), a randomised, double-blind, placebo-controlled phase III trial. We investigated alterations in circadian rhythm in patients with *BRCA* wild-type ovarian cancer undergoing maintenance therapy with rucaparib or receiving a placebo. Our study aimed to characterise and monitor these circadian changes and explore potential correlations with treatment-related side effects (Fig. 1).

**Fig. 2:**
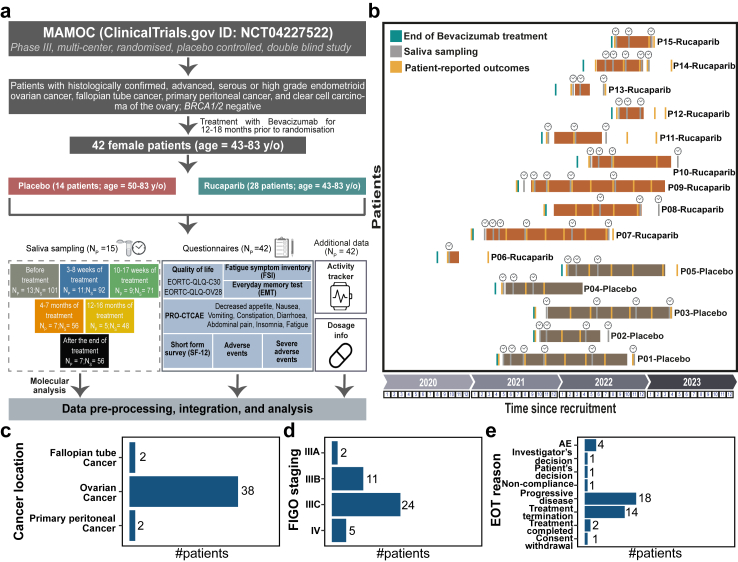
**Study design, patient recruitment timeline, and clinical characteristics of the cohort. a**. Schematic representation of the overall study design. 42 patients with high-grade ovarian cancer (14 placebo, 28 rucaparib) were recruited. Saliva samples from 15 patients were collected at multiple time points throughout treatment (N_P_ = number of patients; N_S_ = number of samples). All the patients completed various questionnaires covering topics such as quality of life and adverse event grading. Additional data on dosage changes, treatment interruptions, and activity tracker metrics were collected. **b.** Overview of patient recruitment timeline and collection of PRO for those who provided saliva samples. The time points for saliva sample collection (for circadian rhythm analysis) and PRO (EORTC-QLQ-C30, FSI, etc.) are shown. Green bars indicate the end of previous bevacizumab therapy, grey bars mark saliva sample collection for molecular circadian analysis, and yellow bars indicate questionnaire completion during treatment. Patient IDs (P01–P15) are colour-coded: placebo (brown) and rucaparib (orange). **c-e.** Descriptive analysis of cancer characteristics. The bar chart displays the clinical classification of 42 patients based on **c**. ovarian cancer type, **d.** cancer severity according to FIGO staging, and **e.** reasons for treatment discontinuation (EOT). Statistical analysis was performed using Fisher's exact test.

A total of 42 patients were recruited for the MAMOC trial and randomised into two groups: rucaparib (N_P_ (#patients) = 28) and placebo (N_P_ = 14) (Fig. 2a). All patients completed questionnaires at different treatment stages and wore an activity tracker. A subgroup of 15 patients (Circadian Cohort: 5 Placebo, 10 Rucaparib) provided saliva for the molecular profiling of their circadian clock (Fig. 2a). The mean age at diagnosis (Mean ± SD) was 66.29 ± 9.34 years for the placebo-treated group and 62.93 ± 10.79 years for the rucaparib-treated group (Table 1). All patients had undergone previous anti-cancer treatments (Table 2).

**Table 1:** Characteristics of the study cohort.

Characteristics	Total patients (N_P_ = 42)	
	Placebo (N_P_ = 14)	Rucaparib (N_P_ = 28)
Age at diagnosis (years, Mean ± SD)	66.29 ± 9.34	62.93 ± 10.79
Cancer type		
Ovarian cancer	14	24
Fallopian tube cancer	0	2
Primary peritoneal cancer	0	2
FIGO staging		
IIIA	1	1
IIIB	3	8
IIIC	8	16
IV	2	3
Lymph node status (TNM staging)		
N0	5	12
N1	5	9
Nx	4	7

N_P_, #patients; FIGO, International Federation of Gynaecology and Obstetrics; TNM, Tumour, Node, Metastasis.

**Table 2:** Summary of previous anti-cancer therapies, surgeries, and chemotherapy in the MAMOC cohort.

	Total patients (N_P_ = 42)	
	Placebo (N_P_ = 14)	Rucaparib (N_P_ = 28)
**Previous anti-cancer treatments**		
Surgery + adjuvant CTx	13	24
Surgery + perioperative CTx (neoadjuvant + adjuvant)	1	4
**Previous surgery performed for OC**		
Yes	12	25
No	2	3
**Primary resection performed?**		
Yes	13	23
No	1	5
**Previous surgery outcome**		
Tumour-free	7	15
Not tumour-free	7	10
No outcome	–	3
**Re-surgery performed for OC**		
Yes	1	6
No	13	22
**Chemotherapy components in first-line**		
Carboplatin	14	28
Paclitaxel	14	28
**Chemotherapy response**		
Complete response	8	13
Partial response	3	4
Stable disease	3	11

N_P_, #patients; OC, Ovarian cancer.

Non-invasive saliva sampling was used to monitor circadian rhythms before, during and after treatment. Patients also completed quality-of-life questionnaires (EORTC-QLQ, EORTC-OV28), fatigue symptom inventory (FSI), Everyday memory tests (EMT), and health status (short-form survey, SF-12), assessments, alongside recording adverse events (CTCAE) (Fig. 2b). Activity tracker data provided insights into physical activity. This phase III trial also tracked changes in drug dosage and therapy duration, which ranged from 3 months to one year, ensuring a comprehensive overview of the treatment course and its impacts.

All 42 patients had previously received bevacizumab therapy within a timeframe of 12–18 months with treatment interruptions (Table S2), and were either tumour-free or at a stable disease state prior to PARPi therapy. Rucaparib (or placebo) maintenance therapy duration among the Circadian Cohort varied, with some patients receiving treatment for 3–4 months, while others continued for up to one year (Fig. 2b).

Saliva samples were collected throughout the treatment period for molecular analysis, with baseline samples taken before PARPi therapy initiation. Some patients in the rucaparib group also provided saliva samples after completing therapy but before starting any new anti-cancer treatment, forming the “rucaparib after the end of therapy (Rucaparib_A_EOT)” group. The MAMOC Cohort included patients with ovarian cancer (N_P_ = 38), fallopian tube cancer (N_P_ = 2) and primary peritoneal cancer (N_P_ = 2) (Fig. 2c). Most of the patients from the MAMOC Cohort were diagnosed with FIGO stage IIIC (N_P_ = 24), followed by IIIB (N_P_ = 11), IV (N_P_ = 5), and IIIA (N_P_ = 2) (Fig. 2d; Table 1). Despite the known benefits of PARPi as maintenance therapy for high-grade tumours, the treatment had to be terminated among some patients due to disease progression (N_P_ = 9), adverse events (AE) or severe adverse events (SAE) (N_P_ = 4) (Fig. 2e).

**Fig. 3:**
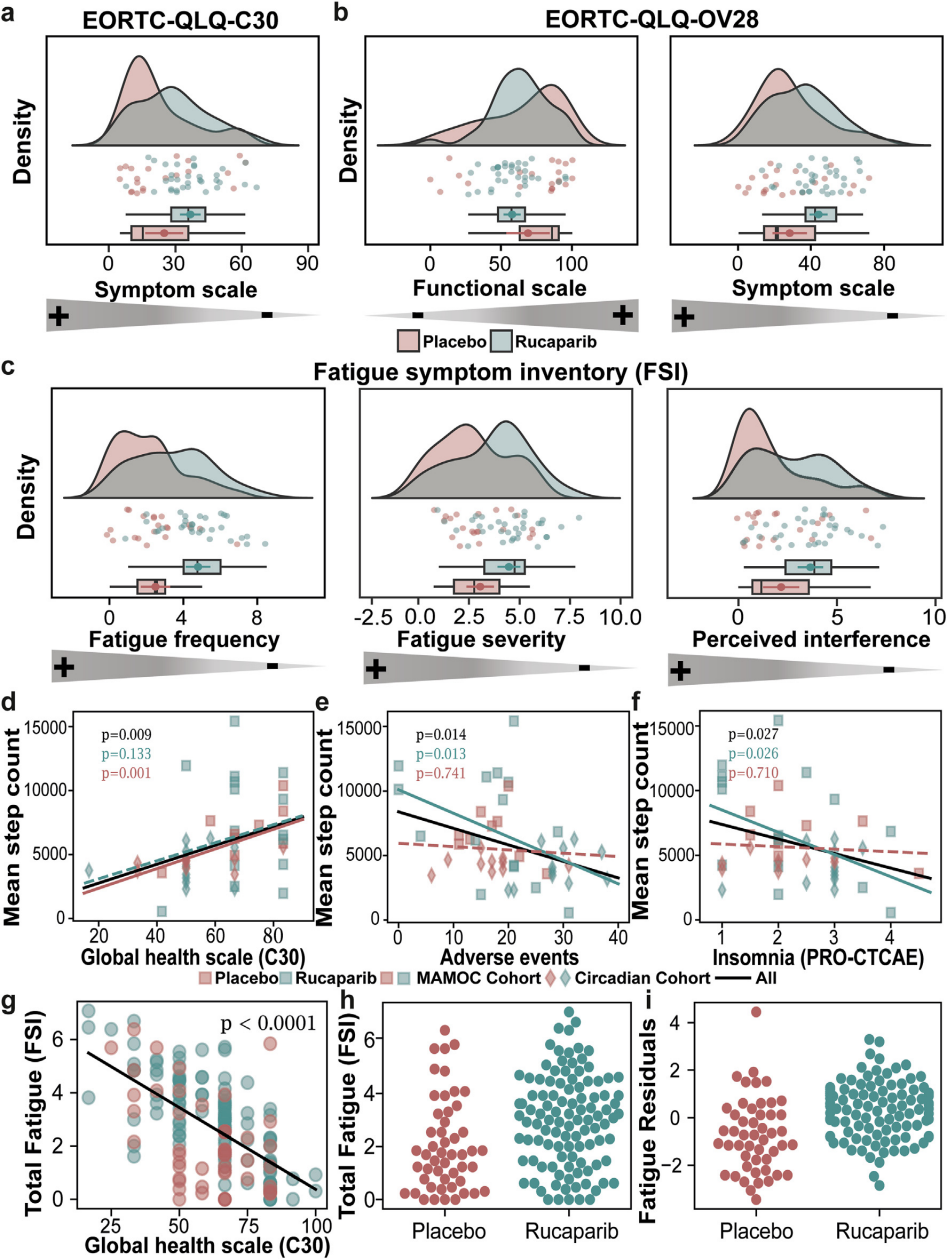
**Descriptive analysis of patientreported outcomes(PRO)and correlations with activity tracker data. a-c.** PRO. Patients in the placebo and rucaparib groups completed questionnaires evaluating quality of life and adverse events. “+" indicates fewer negative effects, and "–" represents more negative effects. The density plot shows the distribution of the full MAMOC cohort (14 placebo, 28 rucaparib), while the jitter plots and boxplots represent the Circadian cohort (5 placebo, 10 rucaparib). **a-b.** The EORTC-QLQ-C30 and EORTC-QLQ-OV28 showed worsened physical functioning and increased severe symptoms in the rucaparib group. **c.** The FSI revealed more severe fatigue in the rucaparib group. Statistical analysis was performed using Wilcoxon rank-sum test with BH correction; error bars are 95% confidence intervals. **d-f.** Correlations between activity and PRO. Step count recorded over one month, was plotted against PRO data for the same period. **d.** Step count correlated positively with the Global health scale. **e.** Step count decreased with higher PRO-CTCAE scores. **f.** Step count decreased with higher insomnia scores. OLS regression was used for the fitting, and *p*-values were obtained for each correlation. **g-i**. Global health scale correlates negatively with fatigue. OLS regression was used in panel g, Wilcoxon rank-sum test was used in panels h and i.

We assessed descriptive parameters (median, IQR) and performed a Kolmogorov–Smirnov test using PRO items to determine whether the Circadian Cohort is a good representation of the MAMOC cohort for both the placebo and rucaparib groups (Table 3). QoL (quality of life) parameters, including the Functional Scale, Symptom Scale, and PRO-CTCAE items such as nausea and insomnia, showed a similar distribution between the placebo group (MAMOC Cohort vs. Circadian Cohort) and the rucaparib group (MAMOC Cohort vs. Circadian Cohort). In particular, quality of life assessments, based on the EORTC questionnaire, revealed a decline in the rucaparib group compared to the placebo group in the MAMOC Cohort (Symptom scale (QLQ-C30): *p* = 0.03; Symptom scale (OV28): *p* = 0.05; Fig. 3a and b) and in the Circadian Cohort (Symptom scale (QLQ-C30): *p* = 0.006, q = 0.015; Functional scale (OV28): *p* = 0.023, *q* = 0.04; Symptom scale (OV28): *p* = 0.003, *q* = 0.01; Fig. 3a and b). Furthermore, the rucaparib group depicted increased AE such as fatigue in the MAMOC Cohort (Fatigue frequency: *p* = 0.003, *q* = 0.05; fatigue severity: *p* = 0.018; fatigue perceived interference: *p* = 0.005, *q* = 0.05; Fig. 3c) and in the Circadian Cohort (Fatigue frequency: *p* = 0.0001, *q* = 0.001; fatigue severity: *p* = 0.002, *q* = 0.010; fatigue perceived interference: *p* = 0.0006, *q* = 0.015; Fig. 3c). Furthermore, deteriorated physical and mental health, along with a higher incidence of adverse events like nausea, insomnia, and abdominal pain were observed in rucaparib compared to the placebo group (Figure S1). Most patients in the rucaparib group showed AE (Tables S3–S4), with grade 3 events like anaemia and elevated alanine aminotransferase levels being therapy-related (Table S3). SAEs, including grade 3 or higher, anaemia and acute pancreatitis, were also observed during therapy (Table S5). During this study, one of the patients (P15-Rucaparib) passed away; however, the cause of death was unrelated to the ongoing therapy.

**Table 3:** Descriptive statistics and Kolmogorov–Smirnov test results for pro items: comparison of circadian cohort and MAMOC cohort by treatment group.

PRO item	Cohort	Group	Median	IQR	Q1	Q3	*p*-value
Functional scale (from C30)	MAMOC	Placebo	76.67	26.67	57.78	84.44	0.94
	Circadian		75.79	37.58	51.11	88.69	
	MAMOC	Rucaparib	68.89	31.11	55.56	86.67	0.04
	Circadian		55.56	32.22	44.44	76.67	
Symptom scale (from C30)	MAMOC	Placebo	17.95	17.95	12.82	30.77	0.99
	Circadian		16.67	25	10.26	35.26	
	MAMOC	Rucaparib	28.20	23.08	15.38	38.46	0.11
	Circadian		33.33	14.42	26.92	41.35	
Global health scale (from C30)	MAMOC	Placebo	66.67	16.67	50.00	60.67	0.98
	Circadian		58.33	16.67	50.00	60.67	
	MAMOC	Rucaparib	66.67	33.33	50.00	83.33	0.03
	Circadian		50.00	25.00	41.67	66.67	
Functional scale (from OV28)	MAMOC	Placebo	66.67	38.10	47.62	85.71	0.38
	Circadian		85.45	39.02	51.46	90.48	
	MAMOC	Rucaparib	61.90	27.51	48.68	76.19	0.35
	Circadian		61.90	19.05	47.62	66.67	
Symptom scale (from OV28)	MAMOC	Placebo	27.78	23.80	17.86	41.67	0.64
	Circadian		22.56	26.80	14.04	40.84	
	MAMOC	Rucaparib	33.33	28.75	20.37	49.12	0.29
	Circadian		40.74	18.57	34.21	52.78	
Memory problem (from EMT)	MAMOC	Placebo	1.54	0.78	1.31	2.09	0.99
	Circadian		1.65	0.91	1.25	2.15	
	MAMOC	Rucaparib	1.54	1.07	1.16	2.23	0.16
	Circadian		1.85	0.88	1.35	2.23	
Fatigue frequency (from FSI)	MAMOC	Placebo	2.00	2.00	1.00	3.00	0.82
	Circadian		2.50	1.50	1.50	3.00	
	MAMOC	Rucaparib	3.00	2.50	2.00	4.50	0.10
	Circadian		4.50	2.63	2.88	5.50	
Fatigue severity (from FSI)	MAMOC	Placebo	2.50	2.75	1.25	4.00	0.75
	Circadian		2.88	2.25	1.75	4.00	
	MAMOC	Rucaparib	3.88	2.75	2.25	5.50	0.29
	Circadian		4.50	2.25	3.00	5.25	
Perceived interference (from FSI)	MAMOC	Placebo	1.14	2.11	0.32	2.43	0.75
	Circadian		1.23	2.82	0.61	3.43	
	MAMOC	Rucaparib	2.43	3.14	1.00	4.14	0.19
	Circadian		3.50	2.26	2.14	4.40	
PCS (from SF-12)	MAMOC	Placebo	37.12	12.08	30.07	42.15	0.95
	Circadian		36.45	10.48	29.66	40.14	
	MAMOC	Rucaparib	36.79	9.05	31.76	40.81	0.33
	Circadian		32.70	9.38	30.38	39.76	
MCS (from SF-12)	MAMOC	Placebo	47.02	10.33	40.52	50.85	0.99
	Circadian		46.77	9.88	41.14	51.02	
	MAMOC	Rucaparib	46.89	11.97	38.56	50.53	0.36
	Circadian		41.39	14.69	35.60	50.29	
Fatigue (from PRO-CTCAE)	MAMOC	Placebo	2.00	1.00	1.50	2.50	0.45
	Circadian		2.00	0.50	2.00	2.50	
	MAMOC	Rucaparib	2.50	1.00	2.00	3.00	0.20
	Circadian		3.00	1.38	2.13	3.50	
Insomnia (from PRO-CTCAE)	MAMOC	Placebo	2.00	1.00	2.00	3.00	0.99
	Circadian		2.00	1.00	2.00	3.00	
	MAMOC	Rucaparib	2.50	2.50	1.00	3.50	0.62
	Circadian		3.00	1.00	2.00	3.00	
Nausea (from PRO-CTCAE)	MAMOC	Placebo	1.00	1.00	1.00	2.00	0.99
	Circadian		1.00	1.00	1.00	2.00	
	MAMOC	Rucaparib	1.00	2.00	1.00	3.00	0.056
	Circadian		2.50	2.50	1.00	3.50	
Vomiting (from PRO-CTCAE)	MAMOC	Placebo	1.00	0.00	1.00	1.00	0.99
	Circadian		1.00	0.00	1.00	1.00	
	MAMOC	Rucaparib	1.00	0.00	1.00	1.00	0.99
	Circadian		1.00	0.00	1.00		
Abdominal pain (from PRO-CTCAE)	MAMOC	Placebo	1.00	1.00	1.00	2.00	0.99
	Circadian		1.00	1.00	1.00	2.00	
	MAMOC	Rucaparib	1.67	1.67	1.00	2.67	0.31
	Circadian		2.50	2.00	1.00	3.00	
Decreased appetite (from PRO-CTCAE)	MAMOC	Placebo	1.00	0.00	1.00	1.00	0.98
	Circadian		1.00	0.50	1.00	1.50	
	MAMOC	Rucaparib	1.00	1.00	1.00	2.000	0.16
	Circadian		1.50	1.50	1.00	2.50	

IQR, Interquartile Range; Q1, first quartile; Q3, third quartile; PCS, Physical Component Summary; MCS, Mental Component Summary.

In addition, we further examined the impact of treatment on daily activity levels and QoL of the patients using activity tracker data and PRO assessments. We used the activity tracker data to investigate a potential correlation between physical activity (mean number of steps taken per day over one month) and PRO data (Fig. 3d–i). Daily activity was analysed for the same month during which PRO data was collected. The mean step count correlated positively with the Global health scale, and negatively with adverse events (PRO-CTCAE score) and insomnia (Fig. 3d–i). The correlation between daily steps and AE was strongly negatively correlated in the rucaparib group (β = −0.48, 95% CI [−0.85, −0.11]; *p* = 0.013) vs. the placebo group (β = −0.081, 95% CI [−0.59, 0.43; Table S6]; *p* = 0.74). These differences in precision, as reflected by CI widths, suggest a stronger and more reliably estimated effect in the rucaparib group. The relatively narrow CI in the rucaparib group (width ≈ 0.74) indicates a moderate sample size with reasonably high precision, strengthening confidence in the observed negative association. In contrast, the wider CI in the placebo group (width ≈ 1.02) reflects greater uncertainty around the estimate. The analysis hints at a steeper decline in step count with stronger adverse events, as well as insomnia in the rucaparib group as compared to the placebo group. This suggests that insomnia, which is influenced by circadian rhythmicity, is associated with global health, side effects, and physical activity.

### Characterisation of circadian clock-related changes in patients treated with rucaparib vs. placebo

**Fig. 4:**
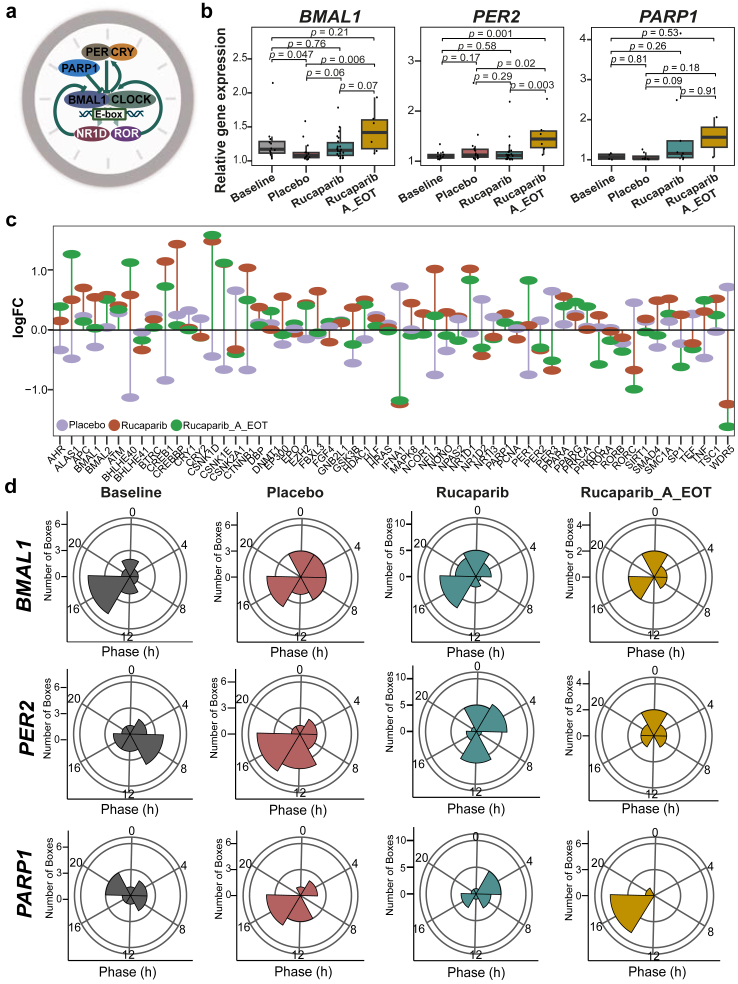
**Rucaparib therapy resulted in alterations of core-clock gene expression and its circadian properties. a.** Graphical representation of the interaction between the core-clock network, especially *BMAL1* and *PARP1*. **b.***BMAL1* showed significantly lower expression in Placebo vs. Baseline, and higher expression in Rucaparib_A_EOT vs. Placebo. Whereas, *PER2* showed significantly higher expression in Rucaparib_A_EOT vs. other groups. *PARP1* expression was higher in Rucaparib and Rucaparib_A_EOT groups, while no significant differences were observed. Outliers were defined as data points falling outside 1.5 times the interquartile range (IQR) from the first (Q1) and third (Q3) quartiles, following standard statistical practices. Individual data points are overlaid as jittered dots. Statistical test used: Wilcoxon rank-sum test (5 placebo, 10 rucaparib). **c.** Lollipop plot represents the changes in the expression of the NCRG genes due to Rucaparib therapy or no therapy (Placebo) vs. the Baseline (N_S_ = 84). **d.** Acrophase bin plot represents the changes in phase (first peak expression) in *BMAL1*, *PER2*, and *PARP1* genes due to Rucaparib therapy vs the Baseline (5 placebo, 10 rucaparib).

As described in the introduction, disruptions in the circadian clock are linked to cancer, and recent evidence suggests a direct connection between PARP1 and the core clock network (Fig. 4a). Moreover, the DNA repair process, regulated by PARP1, is influenced by the circadian clock. PARP1 plays a crucial role in linking feeding cycles with the mammalian circadian system. The activity of PARP1 oscillates in a daily manner in the liver and is regulated by feeding, with PARP1 binding to poly (ADP-ribosyl)ation of (PARylation) CLOCK at the beginning of the light phase.^54^ This interaction affects CLOCK-BMAL1 binding to DNA and alters the phase of their interaction with PER and CRY repressor proteins, ultimately influencing CLOCK-BMAL1-dependent gene expression and the entrainment of peripheral circadian clocks to feeding–fasting cycles.^55^ Furthermore, PARP1 forms a complex with PRMT6 and CRL4B that co-occupies the core clock gene *PER3* promoter, interfering with circadian rhythm oscillation.^56^ This led us to hypothesise that rucaparib therapy might alter the circadian clock and correlate with the observed adverse events. *BMAL1* and *PER2* are core elements of the circadian clock. These genes are crucial to generate the 24-h circadian rhythms, which then regulate the time of biological processes, including metabolism,^57^ hormone release,^58^ and cellular repair mechanisms.^59^ By analysing *BMAL1* and *PER2* expression, we aim to understand how rucaparib therapy influenced circadian rhythms, potentially influencing treatment efficacy, toxicity, and side effects. To explore this, we analysed the average gene expression changes of *BMAL1* and *PER2*, key components of the circadian clock, and *PARP1* (Fig. 4b). *BMAL1* expression decreased in the rucaparib group as compared to the baseline. Interestingly, *PER2* expression was significantly increased after the end of rucaparib therapy vs. baseline (*p* = 0.0014, q = 0.01). Conversely, *PARP1* expression was higher in the rucaparib group vs. baseline. We used a multiplex analysis with Nanostring to further examine a set of 800 genes, including core-clock and clock-regulated genes (previously defined network of clock-regulated genes, NCRG)^42^ (Fig. 4c). Several genes displayed an opposite expression pattern during therapy, which reverted to their original state after the rucaparib treatment ended. Notably, *NFIL3*, a transcription factor crucial for regulating immune function, circadian rhythms, metabolism, and cell survival,^20^^,^^60^ was significantly downregulated in the placebo group compared to baseline levels (logFC = −0.753, 95% CI [−1.28, −0.22], *p* = 0.007), but significantly upregulated in the rucaparib group as compared to baseline (logFC = 1.012, 95% CI [0.204, 1.82], *p* = 0.016). The relatively well-bounded confidence intervals in both groups reflect adequate precision, strengthening confidence in the observed bidirectional regulation of *NFIL3* in response to treatment. This dynamic suggests a reversible impact of the rucaparib therapy on gene expression, with *NFIL3* acting as a potential biomarker of treatment response. Moreover, *NFIL3* gene was significantly upregulated in the rucaparib group compared to placebo group (logFC = 1.766, 95% CI [0.634, 2.89], *p* = 0.003). The wide separation from zero and relatively tight confidence interval suggest a robust and well-estimated treatment-related effect on *NFIL3* expression. However, after the termination of rucaparib therapy, this effect was reversed (Fig. 4c). Similarly, *GSK3B*, a transcription factor known as a negative regulator of glucose homoeostasis^61^ and a key player in the phosphorylation of core clock proteins,^62^ was significantly downregulated in the placebo group compared to baseline (logFC = −0.55, 95% CI [−0.94, −0.17], *p* = 0.006), with a relatively narrow confidence interval supporting the precision of this effect. In the rucaparib group, *GSK3B* expression increased during therapy (logFC = 0.369, 95% CI [−0.39, 1.13], *p* = 0.34), though this change was not statistically significant, and decreased following the conclusion of therapy (logFC = −0.245, 95% CI [−0.88, 0.39], *p* = 0.45). These patterns may reflect dynamic, treatment-related modulation of *GSK3B* expression over time. Furthermore, *RORC*, a core-clock component, acts as a transcription factor, and regulates various cancer-related processes such as cell proliferation, immune cell infiltration, and expression of immunomodulators, thereby influencing the formation of metastatic niches.^63^ *RORC* was upregulated in the placebo group vs. baseline (logFC = 0.451, 95% CI [−0.30, 1.20], *p* = 0.24), whereas *RORC* was significantly downregulated in the rucaparib group when compared to the baseline (logFC = −0.673, 95% CI [−1.26, −0.07], *p* = 0.02) (Fig. 4c; Figure S2). Moreover, *RORC* was significantly downregulated in the rucaparib group compared to the placebo group (logFC = −1.12, 95% CI [−1.84, −0.40], *p* = 0.003). Interestingly, *RORC* expression significantly declined after the end of rucaparib therapy compared to baseline (logFC = −0.99, 95% CI [−1.64, −0.34], *p* = 0.004). Together, these consistent and well-estimated effects suggest a robust downregulation of *RORC* in response to rucaparib treatment, persisting even after therapy cessation. Furthermore, these results suggest that rucaparib therapy altered the expression of core clock and clock-regulated genes. These findings suggest that while rucaparib therapy may induce alterations in core-clock and cancer-associated gene expression, some of which return to baseline levels after therapy, others may persist, potentially contributing to ongoing effects on the patient's quality of life and adverse events.

We investigated the impact of rucaparib therapy on other circadian properties, such as amplitude and acrophase, of *BMAL1, PER2*, and *PARP1*. The circadian profile of *BMAL1* varied depending on treatment status. For instance, most baseline samples collected before treatment peaked at 16 h (Fig. 4d), while in the placebo group, peak expression occurred before 16 h. Interestingly, *BMAL1* in the rucaparib group exhibited a bimodal distribution: some patients had peak expression before 8 h, while most reached peak expression after 12 h (Fig. 4d). A similar pattern was observed for *PER2* in the rucaparib group. Samples from patients in the rucaparib group collected during the first 4–17 weeks of treatment showed *BMAL1* peak expression after 12 h, whereas those collected between 6 and 12 months of treatment peaked before 8 h (Figure S2). This bimodal distribution was absent in the placebo group, suggesting that rucaparib therapy significantly alters the circadian clock properties of *BMAL1* and *PER2*.

Given the intricate relationship between circadian rhythms and pharmacological interventions, we further examined the potential influence of concomitant medications on patient outcomes and circadian assessments. All 42 patients, except P05-Placebo, received concomitant medications during therapy, either due to their medical history or to manage AEs (Table S7). Ten patients received corticosteroids at least once during this trial (P03-Placebo, P07-Rucaparib, P11-Rucaparib, P15-Rucaparib, P21-Placebo, P27-Rucaparib, P30-Rucaparib, P31-Rucaparib, P39-Rucaparib, P40-Rucaparib). No saliva samples for circadian analysis were provided by P07-Rucaparib during the period when corticosteroids were taken, thereby minimising potential confounding effects on circadian rhythm assessment. However, since P11-Rucaparib was continuously receiving corticosteroids, the circadian data of this patient may have been influenced by the known effects of corticosteroids on circadian rhythms. Furthermore, descriptive statistical analysis was performed to assess differences between patients who used corticosteroids and those who did not (Table S8). In the placebo group, fatigue severity (*g* = 1.03, 95% CI [0.34, 1.71]; *p* = 0.002, *q* = 0.02) and symptom scale (*g* = 1.18, 95% CI [0.49, 1.88]; *p* = 0.0007, *q* = 0.01) showed a large and significant effect size between corticosteroid and non-corticosteroid groups, while no large effect size was observed in the rucaparib group (Fatigue severity: g = −0.30, 95% CI [−0.73, 1.12]; *p* = 0.19, *q* = 0.39; Symptom scale: *g* = −0.29, 95% CI [−0.72, 0.13]; *p* = 0.08, *q* = 0.26) (Table S8). Notably, the confidence intervals in the rucaparib group remain wide -particularly for fatigue severity (CI width = 1.85)- indicating substantial uncertainty around the effect estimate. This suggests that while a large effect is unlikely, the current data may not be sufficiently powered to detect smaller, yet potentially meaningful, differences between the corticosteroid and non-corticosteroid groups. In comparison, the placebo group showed large effect sizes, but also had relatively wide CIs (e.g., 1.37–1.39), highlighting variability in estimates across both groups and reinforcing the need for cautious interpretation and further validation in larger cohorts.

**Fig. 5:**
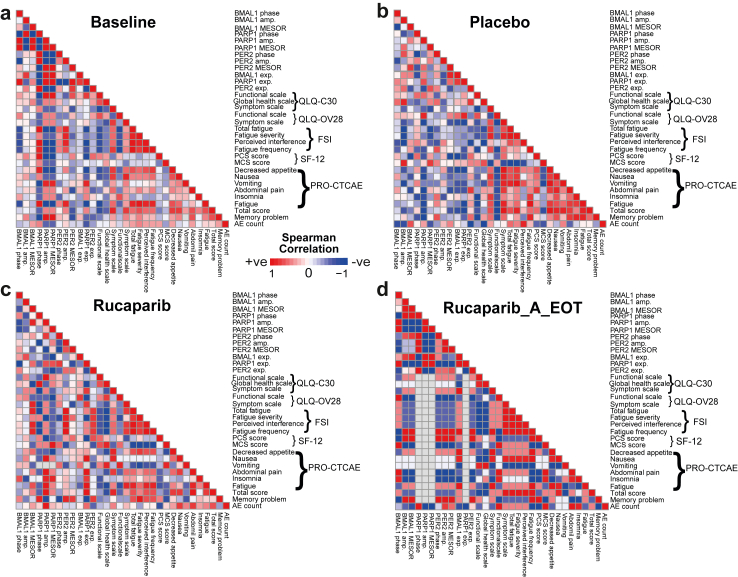
**Alterations of clock properties correlate withPROand adverse events in the rucaparib group.** To investigate potential correlations between *BMAL1*, *PER2*, and *PARP1* gene expression, their circadian properties, and PRO, we performed a Spearman correlation analysis at the patient level (5 placebo, 10 rucaparib). Spearman rank correlation and permutation tests were used to assess the significance of the correlation pairs. **a.** At baseline, *BMAL1* amplitude showed a weak negative correlation with insomnia. **b.** In the placebo group, the correlation between *BMAL1* amplitude and insomnia was slightly positive. **c.** In the rucaparib treatment group, this correlation was strongly negative. **d.** After the end of treatment, the correlation remained negative but was weaker than during treatment.

To determine whether there is a direct link between adverse events and circadian clock characteristics—such as gene expression, amplitude, and acrophase—we conducted a correlation analysis at both the patient level (Fig. 5, Figure S4) and at the saliva kit level (to account for multiple samples collected during treatment; Figures S3 and S5). We found several significant correlations between side effects and specific clock phenotypes, which were specific to the rucaparib group at the patient level. In particular, *PER2* amplitude showed a strong significant positive correlation with memory problems (ρ = 0.883, 95% CI [0.2, 1]; *p* = 0.008) and fatigue frequency (ρ = 0.942, 95% CI [0.33, 1]; *p* = 0.005) in the rucaparib group (Table S9). This correlation remained significant when tested by permutation (Table S10). While in the placebo group, *PER2* amplitude showed a negative correlation with memory problems (ρ = −0.3, 95% CI [−1, 1]; *p* = 0.62) and a weaker positive correlation with fatigue frequency (ρ = 0.102, 95% CI [−1, 1]; *p* = 0.86) (Fig. 5a–d). The wider confidence intervals in the placebo group (CI width = 2) indicate greater uncertainty in these estimates compared to the narrower confidence intervals in the rucaparib group (CI width ≈ 0.8), suggesting more precise and reliable correlations in the rucaparib group. These results indicate that while the rucaparib group shows clear, significant, and precise correlations, the broader confidence intervals in the placebo group highlight the lack of consistent associations, suggesting treatment-specific effects. *BMAL1* MESOR showed a significant strong negative correlation with Global health scale (ρ = −0.821, 95% CI [−1, −0.16]; *p* = 0.02) in the rucaparib group, which remained significant when tested by permutation. *BMAL1* MESOR was negatively correlated with Global health scale in the placebo group (ρ = −0.4, 95% CI [−1, 1]; *p* = 0.50). While, the correlation between *BMAL1* MESOR and Global health scale was positive in the Baseline (ρ = 0.429, 95% CI [−0.40, 0.85]; *p* = 0.18) (Fig. 5a). *BMAL1* relative expression showed a significant positive correlation with Functional scale in the rucaparib group (ρ = 0.785, 95% CI [0.032, 1]; *p* = 0.04), and this significance was observed also after the permutation (Fig. 5). *BMAL1* amplitude showed a strong negative correlation with insomnia in the rucaparib group (ρ = −0.748, 95% CI [−1, 0]; *p* = 0.05) and positive correlation in the rucaparib after the end of therapy (ρ = 0.314, 95% CI [−0.204, 0.705]; *p* = 0.33). Whereas, *BMAL1* amplitude showed a weaker positive correlation in the placebo (ρ = 0.1, 95% CI [−1, 1], *p* = 0.87) and negative correlation in the baseline (ρ = −0.197, 95% CI [−0.913, 0.626], *p* = 0.67) (Fig. 5, Figure S4). Overall, these results suggest that rucaparib treatment significantly modulates *BMAL1* clock properties and its relationship with health outcomes, with stronger and more precise correlations compared to the placebo group and baseline. The narrower confidence intervals in the rucaparib group indicate a more reliable estimate, likely reflecting a sufficient sample size, while the broader intervals in the placebo group point to greater uncertainty and potentially lower precision in that group.

At the box level, *PER2* amplitude showed a strong positive correlation with decreased appetite in the rucaparib group (ρ = 0.814, 95% CI [0.20, 0.98]; *p* = 0.004; Figures S3 and S5, Table S11), which remained significant when tested by permutation (Table S12). In the baseline, the correlation between *PER2* amplitude and decreased appetite was positive (ρ = 0.42, 95% CI [−0.28, 0.87]; *p* = 0.15) while the correlation between the above-mentioned pairs was negative in the placebo group (ρ = −0.07, 95% CI [−0.87, 0.67]; *p* = 0.86; Figures S3 and S5, Tables S11 and S12). Notably, the relatively narrow confidence intervals in the rucaparib group (e.g., CI width ≈ 0.78 for *PER2* amplitude and decreased appetite) indicate more precise estimates and likely reflect a sufficient sample size. In contrast, the broader CIs in the placebo group (e.g., CI width ≈ 1.54) suggest lower precision and potentially smaller sample size or greater variability, underscoring the robustness of the observed associations in the rucaparib group. Furthermore, *PER2* MESOR showed a negative correlation with the Global health scale (ρ = −0.75, 95% CI [−0.95, −0.24]; *p* = 0.01). While in the placebo, *PER2* MESOR showed a weaker negative correlation with the Global health scale (ρ = −0.012, 95% CI [−0.82, 0.89]; *p* = 0.96). These findings suggest that circadian rhythm alterations in the rucaparib group correlate with overall toxicity and specific adverse events, such as fatigue, insomnia, and decreased appetite.

### Dynamic alterations in clock phenotypes across treatment stages

**Fig. 6:**
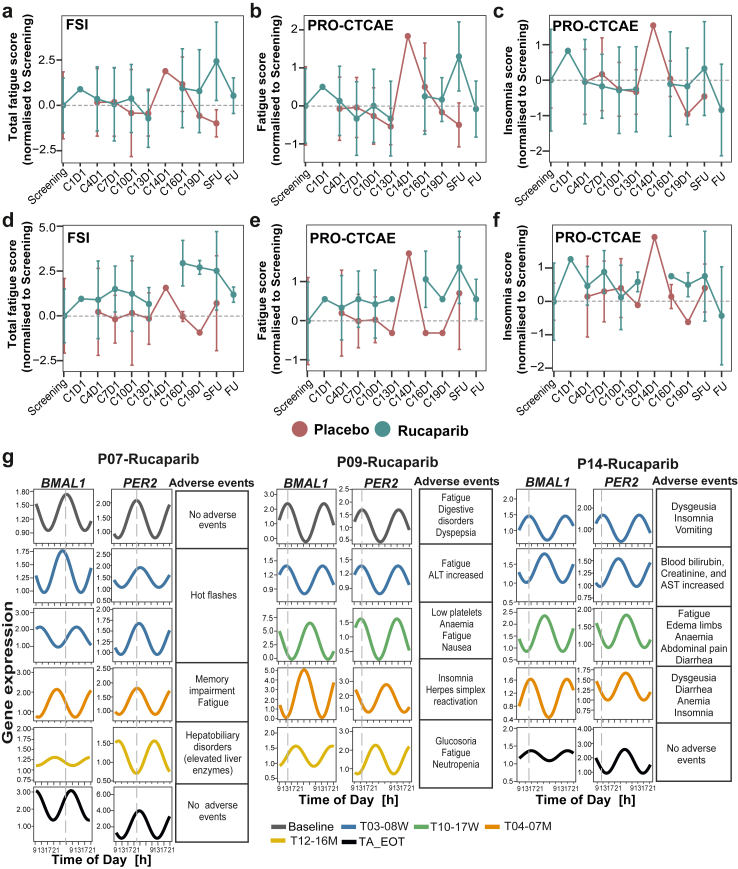
**Circadian dysregulation among patients with rucaparib therapy correlates with reported adverse events. a-f** The rucaparib group showed an increase in adverse events such as fatigue and insomnia **a-c** in the MAMOC cohort (14 placebo, 28 rucaparib), as well as **d-f** in the Circadian cohort (5 placebo, 10 rucaparib) as assessed by a questionnaire. The questionnaire outcomes are visualised across these stages to assess changes over the course of treatment (C: cycle; D: day; SFU: safety follow-up; FU: follow-up; only one patient filled out the questionnaire during C1D1 and C14D1). Error bars are defined as Mean ± SD. **g** The line graphs depict the circadian profile of core clock genes (*BMAL1* and *PER2*) over the treatment timeline for each patient. Adverse events reported by the patients are indicated at relevant time points, highlighting potential associations between circadian profile alterations and side effect occurrences.

**Fig. 7:**
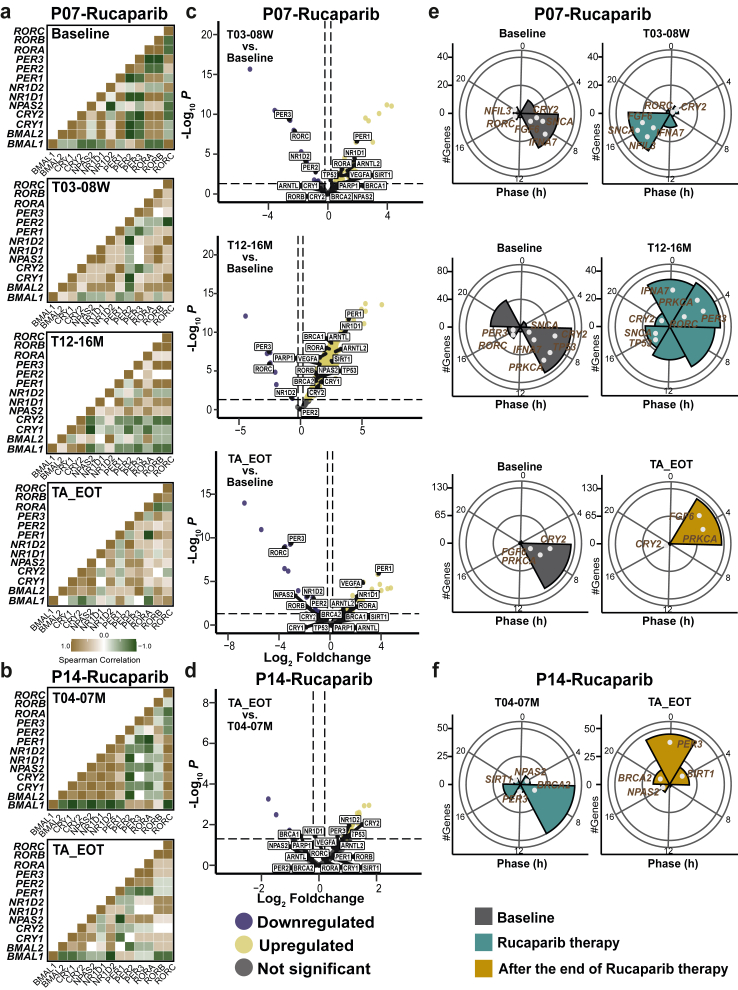
**Circadian dysregulation among patients may reflect the connection strength within the core-clock network and result in the differential regulation and differential rhythmicity of cancer markers. a-b.** Correlation heatmap represents the discrepancies in the strength of the core-clock network due to rucaparib therapy in P07 and P14. **c-d**. Volcano plot represents the differential gene regulation among P07 and P14 patients during rucaparib therapy. **e-f.** Acrophase plot depicts the differential rhythmicity of core-clock and cancer markers due to rucaparib therapy in P07 and P14.

Adverse events, including fatigue and insomnia, increased in the rucaparib group compared to baseline throughout the treatment cycles (from therapy initiation to approximately one year) in the MAMOC Cohort as well as in the Circadian Cohort (Fig. 6a–f). In addition, we evaluated patients' overall functional status during the trial to assess the impact of treatment on physical performance. At screening, most patients in both groups had an ECOG performance status of 0, including 12 patients in the placebo group and 25 patients in the rucaparib group. Over the course of treatment, a decline in ECOG performance status was observed in both groups, with a more pronounced deterioration among patients receiving rucaparib. By Cycle 4 Day 1 (C4D1), the number of patients with ECOG 0 had decreased to 11 in the placebo group and to 15 in the rucaparib group. It is important to note that not all patients had ECOG assessments available at the later cycles, primarily due to treatment discontinuation, disease progression, or loss to follow-up (Table S13). Overall, the quality of life in the rucaparib group deteriorated and the adverse events increased in the rucaparib group when normalised to the screening stage (Figure S6). Given the likely connection between these symptoms and circadian clock disruption, we further examined patients' circadian profiles during therapy. Notably, changes in *BMAL1* and *PER2* amplitude and phase indicate a potential correlation with the severity and frequency of adverse events (Fig. 6g). For P07-Rucaparib and P14-Rucaparib, the treatment was terminated due to disease progression, while for P09-Rucaparib, treatment was terminated due to the end of the study. Similar correlations between circadian profile changes and adverse events were observed in other patients who received rucaparib therapy (Figure S7). Interestingly, such a correlation between the circadian profile of patients and reported adverse events was not observed in the placebo group (Figure S8). These findings prompted us to further investigate the integrity of the core clock network in those patients (Fig. 7a and b). Rucaparib therapy significantly disrupted the correlation patterns among core clock genes, indicating alterations in their regulatory network. Additionally, the strength of the core clock network was assessed in other patients (Figure S9), revealing variations depending on treatment status and therapy stage. This disruption extended to cancer-associated genes, such as *VEGFA*,^64^ *BRCA1*,^65^^,^^66^ *BRCA2*,^66^^,^^67^ *SIRT1,*^68^^,^^69^ and *TP53,*^70^ which are regulated by the circadian clock (Fig. 7c and d). In patients, P07 and P14, these genes exhibited significantly increased expression in response to therapy, particularly *TP53* and *SIRT1* (Fig. 7c and d). The expression levels of various cancer-associated markers also varied depending on whether patients were in the placebo or rucaparib group, as well as the timing of sample collection (Figure S9).

Moreover, a differential rhythmicity analysis was conducted to determine if the circadian properties of the core clock and cancer pathway-associated genes changed significantly during treatment (Fig. 7e–f, Figure S9). Fewer genes displayed differential rhythmicity in the placebo group compared to the rucaparib group. In patient P01-Placebo, most differentially rhythmic genes peaked at 12 h during treatment, compared to 16 h at baseline (Figure S9). For patients in the rucaparib-treated group, several core clock and cancer-associated genes showed differential rhythmicity, especially with long-term rucaparib treatment (Figure S12). For example, in P07-Rucaparib, the core clock gene *CRY2* peaked at 8 h at baseline, 4 h during 8 weeks of treatment, 20 h during 12 months of treatment, and 16 h after therapy ended (Fig. 7e). Genes like *SIRT1, BRCA1, BRCA2*, and *TP53* also exhibited differential rhythmicity in the rucaparib group (Figure S9). These results suggest that rucaparib therapy not only disrupts the core clock machinery, but also impacts key pathways associated with cancer progression.

### Using circadian profiles to predict treatment toxicity outcomes

**Fig. 8:**
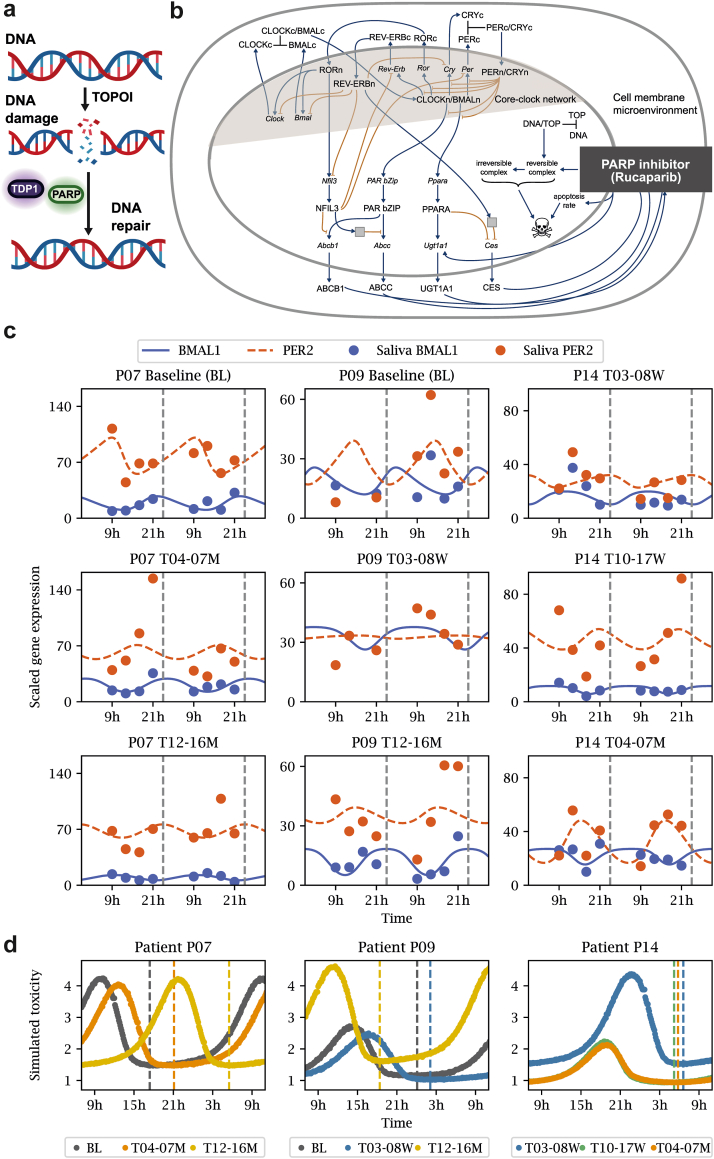
**In silico simulations show an effect of different core clock phenotypes in daily drug toxicity across treatment cycles.** Toxicity profiles have been generated using a previously established model fitted to another drug with a similar mechanism of action also interfering with DNA repair machinery, for different dosing schedules.^46^**a.** Graphical representation of PARP1's mode of action. **b.** A previously published mathematical model for predicting drug toxicity targeting the DNA repair pathway and cell cycle duplication. **c**. Circadian profile of *BMAL1* and *PER2* genes among P07, P09, and P14 based on the gene expression normalised to the model's scale (dots) and the corresponding model fit (line). **d.** Based on the fitted expression profiles, the model predicted the level of rucaparib drug toxicity. Different curves represent specific drug toxicity profiles which result from alterations in circadian rhythms. Marked with dashed lines are the predicted treatment times with minima in toxicity.

PARP1 is essential for DNA repair, catalysing poly (ADP-ribosyl)ation (PARylation) of target proteins in response to DNA strand breaks. It plays a critical role in the repair of single-strand breaks (SSBs) through the base excision repair (BER) pathway. When DNA damage occurs, PARP1 rapidly detects SSBs, recruits repair proteins, and facilitates DNA repair to maintain genomic integrity.^71^ PARP1's repair function is closely linked to topoisomerase I (TOP1), which resolves DNA torsional strain during replication and transcription.^72^ When TOP1 generates transient single-strand nicks, PARP1 assists in resolving these breaks. If TOP1 cleavage complexes (TOP1cc) become trapped, PARP1 helps initiate their removal to prevent replication fork collapse and the conversion of SSBs into more deleterious double-strand breaks (DSBs).^72^ Interestingly, both PARP1^54^^,^^56^ and TOP1^73^^,^^74^ exhibit circadian oscillations, linking DNA repair efficiency to the circadian clock (Fig. 8a).

PARP inhibitors (PARPi) exploit this dependency by trapping PARP1 on damaged DNA, preventing its dissociation and leading to persistent DNA damage. In cancer cells with homologous recombination (HR) defects—such as *BRCA1/2*-mutant tumours—PARP inhibition induces synthetic lethality by converting unrepaired SSBs into lethal DSBs that cannot be efficiently repaired.^75^

Additionally, the transporters and enzymes involved in the metabolism of rucaparib directly interact with the core-clock network (Figure S10). Given the observed side effects, we explored whether the timing of treatment administration could mitigate drug toxicity. We employed a mathematical model developed by our group, which originally focused on DNA repair machinery in the context of Irinotecan, to predict toxicity based on circadian profiles of *BMAL1* and *PER2* (Fig. 8b).^46^ Both Irinotecan and PARPi target critical elements of the DNA damage response pathway: Irinotecan induces DNA damage by inhibiting TOP1, resulting in single-strand breaks (SSBs), while PARP1 is crucial for repairing these SSBs. Effectively, the toxicity of both drugs results from an increase in SSBs, which, as markers of unsuccessful DNA replication, induce cell death. Thus, the model, designed for Irinotecan, can be applicable to PARPi due to their shared role in DNA damage and repair processes.

Patients were advised by their clinicians to take their medication twice daily with a 12-h gap. To assess the impact of treatment timing on toxicity, we analysed variations in circadian rhythms across treatment stages (Fig. 8c–d, Figure S11). The model was parameterised by (i) a fit of gene expression dynamics and (ii) the apoptosis dynamics of a cell culture in response to DNA-duplication interference at different times of the circadian rhythm. In our previously published model, the toxicity variable models the apoptosis, measured as Area Under the Curve (AUC) over the first 48 h of treatment, following the application of Irinotecan to the cell culture at different times of their circadian rhythm.^46^ The toxicity curve hence summarises the expected toxicity accumulated over two days when starting treatment at a particular time of the day for each patient. For fitting the patient data, we freed the parameters involved in *PER2* or *BMAL1* gene expression and the proteins influencing these genes, and fitted the network to the saliva gene expression profiles (Fig. 8c). The circadian profiles allow the model to predict toxicity. We shifted the profiles of circadian degradation and apoptosis of the PK-PD dynamics of the original model according to each patient's simulated phase of *BMAL1* expression. Toxicity profiles change for each patient with treatment progress (Fig. 8d, Figure S11). While the model assumes relatively constant drug availability, patients have a peak concentration in the blood sometime after intake.^76^ The more the maximal blood concentrations overlap with the predicted peak of toxicity, the stronger we would expect the side-effects to be, while a minimal overlap (drug intake around the dashed line) might minimise side-effects. For instance, for P07-Rucaparib, the peak of predicted toxicity moves to later and later times with treatment progression, while for P14-Rucaparib the peak of toxicity advances in phase with treatment progression. Moreover, in patient P07-Rucaparib, there was a notable increase in adverse events at 12 months of treatment, accompanied by a significant upregulation of both cancer-related and clock-related genes compared to baseline. Additionally, the toxicity curve for this patient also showed a marked shift, suggesting a possible link between these factors. In case of patient P14-Rucaparib, there was an overlap in the toxicity curves observed at 10–17 weeks of treatment and 4–7 months of treatment. Additionally, certain adverse events, such as anaemia and insomnia, persisted across both stages in P14-Rucaparib, indicating a continuation of these side effects over time. These variations may explain differences in toxicity profiles among patients, potentially leading to varying side effects. To investigate whether toxicity outcome parameters correlate with PRO items, such as QoL or AEs, we performed a Spearman correlation analysis (Figure S12). The analysis revealed a negative association between AE count and toxicity parameters predicted by the mathematical model, including average toxicity (ρ = 0.465, 95% CI [0.057, 0.780]), toxicity relative amplitude (ρ = 0.314, 95% CI [−0.204, 0.705], *p* = 0.13), and toxicity absolute amplitude (ρ = 0.410, 95% CI [0.020, 0.722]). The negative correlation between AE count and both average toxicity (*p* = 0.02) and toxicity absolute amplitude (*p* = 0.049) was statistically significant. In contrast, a positive correlation was observed between toxicity phase and AE count (ρ = −0.347, 95% CI [−0.67, 0.049], *p* = 0.10). These findings suggest that model-predicted toxicity dynamics are meaningfully associated with adverse event burden. The relatively narrow confidence intervals for the significant correlations (e.g., CI width ≈ 0.72 for average toxicity and ≈0.70 for absolute amplitude) indicate reasonably precise estimates, supporting the reliability of these associations. In contrast, the broader CI for toxicity phase (CI width ≈ 0.72) and lack of statistical significance suggest less certainty in this relationship. These findings suggest that circadian disruptions influence drug toxicity, contributing to increased adverse events, and highlight the potential for treatment timing adjustments to mitigate toxicity-related side effects (Figure S12).

**Fig. 9:**
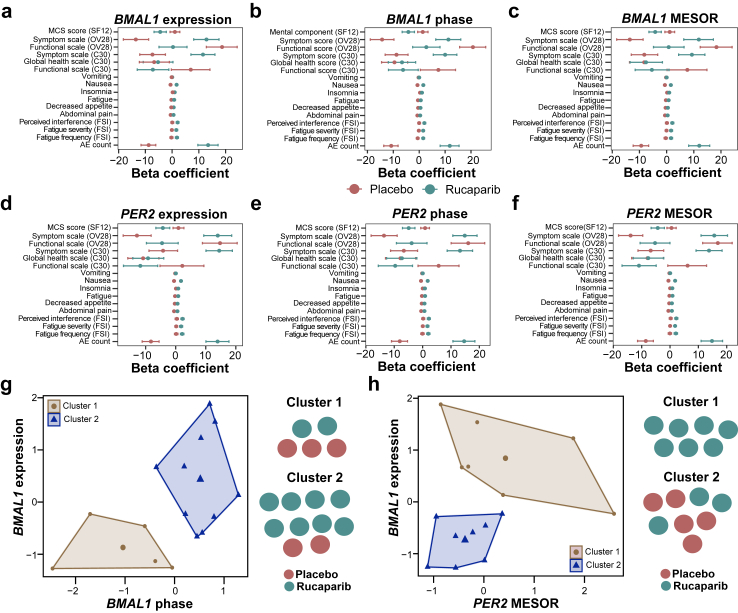
***BMAL1* and *PER2* circadian properties influencePROand can be used to stratify patients into treated and untreated groups. a-f** OLS with bootstrapping analyses demonstrates the relationships between *BMAL1* and *PER2* circadian properties and PRO such as adverse events and quality of life. The error bar represents 95% CI (5 placebo, 10 rucaparib). **g-h** Unsupervised k-means clustering based on *BMAL1* gene expression and *PER2* MESOR identifies distinct patient groupings between the placebo and rucaparib groups (5 placebo, 10 rucaparib).

Our findings underscore the influence of circadian rhythms on drug toxicity and side effects, suggesting that treatment timing adjustments could help manage these effects. The strong interaction between the circadian properties of *BMAL1* and *PER2* with PRO and adverse events was evident (Fig. 5). However, it remains unclear if circadian features of *BMAL1* and *PER2* (such as amplitude, MESOR, and phase) can predict outcomes like adverse events or quality of life. To explore this, we conducted multiple regression analyses using Baseline as the control model (Fig. 9a–f; Figure S13a and b). In the placebo group, *BMAL1* expression, *BMAL1* circadian properties (amplitude, phase, MESOR), *PER2* expression, and *PER2* circadian properties (amplitude, phase, MESOR) negatively correlated with nausea: *BMAL1* expression (β = −0.58, 95% CI [−0.84, −0.33]; *p* < 0.0001), *BMAL1* phase (β = −0.67, 95% CI [−0.94, −0.42]; *p* < 0.0001), *BMAL1* amplitude (β = −0.58, 95% CI [−0.85, −0.32]; *p* < 0.0001), *BMAL1* MESOR (β = −0.63, 95% CI [−0.89, −0.38]; *p* < 0.0001), *PER2* expression (β = −0.55, 95% CI [−0.80, −0.32]; *p* < 0.0001), *PER2* phase (β = −0.56, 95% CI [−0.81, −0.31]; *p* < 0.0001), PER2 amplitude (β = −0.73, 95% CI [−0.95, −0.49]; *p* < 0.0001), *PER2* MESOR (β = −0.56, 95% CI [−0.80, −0.32]; *p* < 0.0001) (Fig. 9a–f; Figure S13a and b). In contrast, in the rucaparib group, these parameters showed positive associations with nausea: *BMAL1* expression (β = 1.69, 95% CI [1.38, 1.98]; *p* < 0.0001), *BMAL1* phase (β = 1.58, 95% CI [1.28, 1.87]; *p* < 0.0001), *BMAL1* amplitude (β = 1.64, 95% CI [1.33, 1.94]; *p* < 0.0001), *BMAL1* MESOR (β = 1.55, 95% CI [1.23, 1.87]; *p* < 0.0001), *PER2* expression (β = 1.76, 95% CI [1.46, 2.05]; *p* < 0.0001), *PER2* phase (β = 1.79, 95% CI [1.48, 2.08]; *p* < 0.0001), *PER2* amplitude (β = 1.76, 95% CI [1.46, 2.05]; *p* < 0.0001), *PER2* MESOR (β = 1.79, 95% CI [1.49, 2.09]; *p* < 0.0001; Table S14). We also evaluated the model fitting between nausea score and various circadian and gene expression parameters. Significant associations were found with *BMAL1* expression (adj. R^2^ = 0.540, *p* < 0.0001), *BMAL1* circadian properties—amplitude (adj. R^2^ = 0.527, *p* < 0.0001), phase (adj. R^2^ = 0.535, *p* < 0.0001), and MESOR (adj. R^2^ = 0.534, *p* < 0.0001)—as well as *PER2* expression (adj. R^2^ = 0.554, *p* < 0.0001). Additionally, *PER2* circadian properties showed strong model fits: amplitude (adj. R^2^ = 0.601, *p* < 0.0001), phase (adj. R^2^ = 0.565, *p* < 0.0001), and MESOR (adj. R^2^ = 0.565, *p* < 0.0001). Furthermore, in the placebo group, *BMAL1* phase and *PER2* amplitude were negatively associated with fatigue: *BMAL1* phase (β = −0.48, 95% CI [−0.69, −0.25], *p* < 0.0001) and *PER2* amplitude (β = −0.33, 95% CI [−0.58, −0.069], *p* = 0.014) (Fig. 9b, Figure S12b; Tables S14–S15). In contrast, these associations were positive in the rucaparib group: *BMAL1* phase (β = 0.61, 95% CI [0.37, 0.83], *p* < 0.0001) and *PER2* amplitude (β = 0.85, 95% CI [0.62, 1.09], *p* = 0.014). The model fit was also significant between fatigue and *BMAL1* phase (adj. R^2^ = 0.337, *p* < 0.0001) as well as *PER2* amplitude (adj. R^2^ = 0.302, *p* < 0.0001) (Tables S14–S15). Together, these results reveal opposing associations between circadian parameters and nausea or fatigue across treatment groups, with robust and significant model fits in the rucaparib group. Moreover, the narrow confidence intervals for key estimates (e.g., CI widths <0.5 for most rucaparib effects) indicate high precision, whereas wider intervals in the placebo group reflect greater variability and likely smaller sample size, underscoring the impact of treatment on the stability and relevance of circadian biomarkers. These findings suggest that core-clock gene expression and its properties are significantly associated with adverse events like nausea and fatigue in both the placebo and rucaparib groups, albeit in opposite directions.

K-means clustering further differentiated between the placebo and rucaparib groups (Fig. 9g and h; Figure S13c and d). *BMAL1* phase and gene expression formed two distinct clusters: Cluster 1 comprised three placebo patients and two rucaparib patients, while Cluster 2 included eight rucaparib patients and two placebo patients (Fig. 9g, Figure S12c). The two patients from the rucaparib group in Cluster 1 were the only patients in the treatment group who had undergone two surgeries before starting chemotherapy and bevacizumab therapy. In contrast, two patients from the placebo group in Cluster 2 were the only placebo patients who were not tumour-free at recruitment. These findings suggest that distinct clinical backgrounds, such as pre-treatment surgical history and tumour status, may influence how patients cluster based on *BMAL1* expression and phase. *BMAL1* gene expression and *PER2* MESOR also formed two distinct clusters: Cluster 1 had seven rucaparib patients, while Cluster 2 included all placebo patients and three additional rucaparib patients, this appears to reflect both the treatment and lymph node status of the patients (Fig. 9h, Figure S13d). Cluster 1, which included patients with lymph node status N0 or N1, consisted entirely of those who received treatment, suggesting a potential association between treatment and specific circadian expression patterns among patients with confirmed lower levels of lymph node involvement. In contrast, Cluster 2 included patients predominantly from the placebo group, along with two rucaparib patients who had an undefined lymph node status (Nx).

In conclusion, our findings highlight the significant role of circadian properties in predicting treatment toxicity and patient outcomes, underscoring the potential benefit of aligning treatment timing with individual circadian rhythms to optimise therapeutic efficacy and minimise adverse effects.

## Discussion

Ovarian cancer remains one of the deadliest malignancies in women, with most cases diagnosed at an advanced stage due to the lack of effective early screening. Despite treatment advances, the prognosis for advanced ovarian cancer remains poor, largely due to limitations of current therapies, such as cytoreductive surgery and platinum-based chemotherapy. The introduction of targeted therapies, particularly PARPi, has improved outcomes, especially in patients with *BRCA* mutations and HRD.^77^ Several phase III trials demonstrate the efficacy of PARPi like olaparib, niraparib, and rucaparib, in significantly improving PFS. However, managing treatment-related toxicity remains a challenge, affecting both patients’ quality of life and therapy adherence.

A promising approach to mitigating toxicity and optimising treatment efficacy is chronotherapy—aligning the timing of drug administration with the patient's circadian rhythms.^78^ The circadian clock, governed by core-clock genes such as *BMAL1* and *PER2*, orchestrates various physiological processes, including metabolism,^79^ cell proliferation,^19^ and responses to medications,^80^ making them a crucial target for improving cancer therapy outcomes. Early studies demonstrated up to five-fold differences in drug tolerability and efficacy based on dosing time in animal models, and more recent evidence confirms similar benefits in cancer patients, particularly in improving the tolerability and efficacy of chemotherapy regimens.^81^ Moreover, randomised trials of cancer chrono-chemotherapy consistently show improved tolerability in 77% of cases, further validating the clinical potential of chronotherapy.^82^ Beyond chemotherapy, radiotherapy and immunotherapy have also shown significant time-dependent effects, with immune checkpoint inhibitors administered in the morning correlating with better survival outcomes across various cancer types.^83^^,^^84^ However, despite the promise of cancer chronotherapy, key challenges remain. Personalised chronotherapy necessitates the integration of biomarkers, such as circadian rhythms and genetic profiles, to tailor treatment schedules to individual patients. Up to 50% of patients with metastatic cancer exhibit disrupted circadian rhythms, which diminishes their responsiveness to chronotherapy and underscores the need to restore circadian functionality as part of treatment.^85^ Indeed, disruption of circadian rhythms has been implicated in cancer progression and treatment response,^23^ suggesting that the timing of PARPi administration could be crucial for maximising therapeutic benefits while minimising adverse effects.

Our study investigated the impact of rucaparib maintenance therapy on circadian rhythms in patients with high-grade ovarian cancer. We observed significant circadian rhythm disruptions in core-clock genes *BMAL1* and *PER2* in patients treated with rucaparib compared to those on placebo. Notably, these disruptions correlated with the severity and frequency of treatment-related adverse events. Specifically, the dysregulation of the *PER2* gene was strongly associated with the number of adverse events, while disruptions in *BMAL1* correlated with the severity of these events. These findings suggest that circadian rhythm dysregulation may play a role in the manifestation of treatment-related toxicity, highlighting the requirement of personalised chronotherapy to improve patient outcomes when the treatment itself induces circadian disruptions. Saliva sampling, and the subsequent analysis of the resulting circadian profiles of core clock genes with computational modelling of circadian drug pharmacokinetics and –dynamics, using the TimeTeller^38^, ^39^, ^40^ methodology, is one promising avenue in this regard.

In addition to circadian considerations, we explored the interaction between PARPi and anti-angiogenic therapies, such as bevacizumab. This combination, which exploits the concept of “contextual synthetic lethality,” has shown promise in enhancing treatment efficacy by inducing tumour hypoxia and increasing DNA damage.^86^^,^^87^ However, the associated toxicity remains a significant challenge. Our findings suggest that integrating chronotherapy with PARPi and bevacizumab therapy could offer a novel strategy to enhance therapeutic efficacy while minimising adverse events. To validate our findings, future studies will need to test this hypothesis in a new cohort. Overall, it seems that circadian disruption greatly contributes to the side-effects of rucaparib therapy.

It is important to notice that the study's design and relatively small sample size limit our ability to fully account for potential stratification variables, leaving the possibility of residual confounding that could not be entirely controlled. However, as an exploratory study, the primary aim was to generate initial insights and identify potential associations that warrant further investigation. Larger, circadian rhythms-stratified cohorts in future studies will be essential to validate these findings and evaluate their broader applicability also for other cancer types. Despite the small sample size, we successfully characterised circadian clock-associated changes in patients with high-grade ovarian cancer undergoing rucaparib therapy. By applying various computational approaches, this exploratory study emphasises the significance of considering the time of day as a factor in minimising toxicity and enhancing drug efficacy. Additionally, correlation analysis revealed a direct relationship with PRO. OLS regression with bootstrapping, applied due to the limited sample size, demonstrated that core clock parameters are significant predictors of PRO. While several associations showed statistical significance, the width of the 95% confidence intervals provides additional insight into the robustness and precision of our estimates. In several cases, especially within the rucaparib group, effect sizes were accompanied by narrow CIs (e.g., *PER2* amplitude and fatigue, ρ = 0.942, 95% CI [0.33, 1]), suggesting consistent trends and relatively stable estimates despite the limited sample size. In contrast, wider CIs observed in the placebo group—often spanning the null—reflect higher variability and likely lower statistical power.

The clinical relevance of our findings is underscored by the strength and consistency of the observed associations between markers and PRO. In particular, significant correlations were found between circadian properties of *BMAL1* and *PER2* with symptoms such as fatigue, insomnia, and nausea. For instance, in the rucaparib group, *BMAL1* expression and *PER2* amplitude showed strong correlations with adverse events, with beta coefficients of 1.69 (95% CI [1.38, 1.98]) and 1.76 (95% CI [1.46, 2.05]), respectively, suggesting a substantial impact on therapy side effects experienced by the patient. Furthermore, the negative correlation between *BMAL1* MESOR and the Global health scale (ρ = −0.821, 95% CI [−1, −0.16]) indicates a clinically meaningful association between circadian rhythm disruptions and global health outcomes. The statistical significance and narrow confidence intervals associated with these estimates reflect reliable and clinically relevant effects, suggesting that circadian biomarkers may indeed serve as valuable predictors of treatment-related toxicity and quality of life in rucaparib-treated patients with ovarian cancer. These findings highlight the potential for incorporating monitoring, for e.g., with non-invasive tools like TimeTeller, into clinical practice to improve patient management and tailor treatment strategies.

The implications of this study are substantial for the field of oncology. By tailoring treatment to align with a patient's circadian rhythms, healthcare providers could reduce the toxicity associated with PARPi, potentially leading to improved quality of life and better adherence to therapy. Moreover, this approach could pave the way for more personalised cancer treatments, where the timing of drug administration is as critical as the choice of the drug itself. This is consistent with previous publications from Francis Lévi's group, which emphasised that “Dosing-time makes the poison”.^88^ Additionally, sex-specific differences in optimal dosing times further support the need for personalisation, with evidence suggesting that men and women may benefit from drug administration at different times of the day.^89^, ^90^, ^91^ Advances in telemonitoring technologies and AI-driven analyses of circadian and tumour clocks offer new tools for refining chronotherapy delivery,^92^ but their integration into routine clinical practice remains a challenge. Future research should focus on overcoming between- and within-patient variability in circadian rhythms and cancer clocks, along with developing user-friendly technologies for real-time circadian monitoring and personalised treatment.

The results reported here have significant medical value for oncology. By identifying the relationship between circadian rhythms and treatment adverse events, we further support the relevance of chrono medicine and our results moreover open potential paths for how healthcare providers can tailor treatment plans to the patient's internal clock. This personalised approach has the potential to reduce the severity and frequency of side effects and thus improve the quality of life of cancer patients.

As the field of chronotherapy continues to evolve, integrating circadian biology into cancer treatment protocols may revolutionise the way we approach cancer care, leading to more effective and less toxic therapies. Ultimately, the growing understanding of circadian biology in cancer treatment presents a unique opportunity to revolutionise oncology, making the timing of interventions as crucial as their nature.

## Contributors

J.S., E.I.B., and A.R. conceptualised the study, acquired the funding, and provided supervision. D.M., J.N., G.R., B.C., J.G., M.K., T.K., O.T., R.W., M.D., M.E., J.S., E.I.B., and A.R. carried out data curation. D.M., J.H., and K.S. carried out formal analysis, software usage, and visualisation. D.M., J.H., N.N., K.S., J.S., E.I.B., and A.R. carried out the investigation. D.M., J.H., N.N., K.S., O.A., J.G., and A.R. contributed in the methodology. D.M., J.N., J.G., E.I.B., and A.R. provided project administration. D.M., J.H., K.S., and A.R. carried out data validation. D.M. and A.R. wrote the original draft of the manuscript. D.M., J.N., E.I.B., and A.R. accessed and verified the obtained data. All authors were involved in the review and editing of the manuscript. All authors read and approved the final version of the manuscript.

## Data sharing statement

Patient data will be provided by the NOGGO (studies@noggo.de) upon request. Any request should be sent to E.I.B. (elena.braicu@charite.de) and A.R. (angela.relogio@medicalschool-hamburg.de) with a detailed description of the research protocol. The NOGGO reserves the right to decide whether to share the data or not. Access will be provided after a proposal has been approved by an independent review committee established for this purpose and after receipt of a signed data-sharing agreement. The code used for data processing and analysis in the study can be shared upon reasonable request. Please contact the corresponding authors for access.

## Declaration of interests

The work in the group of A.R. relative to this manuscript has been financed by the MSH Medical School Hamburg, the Digital Health Accelerator Program of the Charité/BIH Berlin Institute of Health, and by the Dr. Rolf Schwiete Stiftung. A.R. is CEO of TimeTeller GmbH and has granted and pending patents regarding the characterisation of circadian rhythms in saliva for different applications. J.H. is currently at the Leibniz Institute for Resilience Research, Mainz and funded by the Boehringer Ingelheim Foundation. B.C. received honoraria from AstraZeneca and MSD. R.W. received honoraria for lectures by AstraZeneca and GSK, and received travel support from GSK for attending the ESMO conference in 2022. J.S has received funding from Roche Pharma, AstraZeneca, Bayer, Clovis Oncology, GSK, Lilly, Tesaro, and MSD for the MAMOC study; has received consulting fees from Tesaro, Merck, Pfizer, PharmaMar, Clovis Oncology, AstraZeneca, Roche Pharma, GlaxoSmith, MSD, Eisai, Novocure, Oncoinvent, Esai, Tubulis, Immunogen, AbbVie, GSK, Bayer, Vifor Pharma, Hexal AG, Novartis Pharma; has received honoraria from Tesaro, Merck, Pfizer, PharmaMar, Clovis Oncology, AstraZeneca, Roche Pharma; GlaxoSmith, MSD, Eisai, Novocure, Oncoinvent, Esai, Tubulis, Immunogen, AbbVie, GSK, Bayer, Vifor Pharma, Hexal AG, Novartis Pharma; has served a leadership role at NOGGO, AGO, ENGAGe, ENGOT, Deutsche Stiftung für Eierstockkrebs. E.I.B received funding from Clovis Oncology for the MAMOC study; has received honoraria from AstraZeneca, Abbvie, Immunogen, GSK; has received travel support from AstraZeneca; has participated on an Advisory Board for TORL-bio, Tubulis, MSD, GSK, PharmaEnd, Myriad, Immunogen; and is a medical director of the NOGGO. All other authors declare no competing interests.
