# Supplementary material for: Circadian rhythm disruption by PARP inhibitors correlates with treatment toxicity in patients with ovarian cancer and is a predictor of side effects
Source: eBioMedicine. 2025 May 16;117:105764. doi: 10.1016/j.ebiom.2025.105764 (PMC12173083; doi:10.1016/j.ebiom.2025.105764)
Supplement: Supplementary Figures [file mmc1.pdf]

Supplementary File

Circadian Rhythm Disruption by PARP Inhibitors Correlates with Treatment Toxicity in Patients with Ovarian Cancer and is a Predictor of Side Effects

Deeksha Malhan, PhD[1], Janina Hesse, PhD[1,2,3,4], Nina Nelson, PhD[1], Kay Stankov, PhD[5], Jessica Nguyen, MSc[6], Ouda Aboumanify, MSc[7], Josefin Garmshausen, MSc[1,7], Gunther Rogmans, MD[6,8], PD Bastian Czogalla MD[6,9], Jens Gerber MD[6,10], PD Martin Koch MD[11], Tomáš Kupec MD[6,12], Oliver Tomé MD[6,13], Ralf Witteler MD[6.14], Mustafa Deryal MD[6,15], Prof. Michael Eichbaum MD[6,16], Prof. Jalid Sehouli MD[6,17], Prof. Elena Ioana Braicu MD[6,18]*, Prof. Angela Relógio, PhD[1,7,]*

[1]Institute for Systems Medicine and Faculty of Human Medicine, MSH Medical School Hamburg, Hamburg, Germany

[2]Leibniz-Institute for Resilience Research (LIR), Mainz, Germany.

[3]Institute for Quantitative and Computational Biosciences (IQCB), Johannes-Gutenberg University, Mainz, Germany.

[4]Johannes Gutenberg University Medical Center Mainz, Mainz, Germany.

[5]Ainovate GmbH, Frankfurt, Germany

[6]North-Eastern German Society of Gynecological Oncology (NOGGO e.V.), Berlin, Germany

[7]Institute for Theoretical Biology (ITB), Charité—Universitätsmedizin Berlin, Corporate Member of Freie Universität Berlin, Humboldt-Universität zu Berlin, and Berlin Institute of Health, Berlin, Germany

[8]ZAGO- Zentrum für ambulante gynäkologische Onkologie, Krefeld, Germany

[9]Department of Obstetrics and Gynecology, LMU University Hospital, LMU Munich, Munich, Germany

[10]Städtisches Klinikum Dessau, Frauenheilkunde und Geburtshilfe, Dessau, Germany

[11]Department of Gynecology and Obstetrics, Hospital Anregiomed Ansbach, Ansbach, Germany

[12]Department of Obstetrics and Gynecology, University Hospital Aachen, Aachen, Germany

[13]ViDia Christliche Kliniken Karlsruhe, Department of Gynecology and Obstetrics, Karlsruhe, Germany

[14]Universitätsklinikum Münster, Klinik für Frauenheilkunde und Geburtshilfe, Münster, Germany

[15]Center for gynecology, Caritas Klinikum St. Theresia-Saarbruecken, Saarbruecken, Germany

[16]Helios Dr. Horst Schmidt Kliniken Wiesbaden, Department of Gynecology and Obstetrics, Wiesbaden, Germany

[17]Department of Gynecology with Center for Oncological Surgery, Charité - Universitätsmedizin Berlin, Campus Virchow Klinikum, Berlin, Germany

[18]Tumorbank Ovarian Cancer Network, Charité - Universitätsmedizin Berlin, corporate member of Freie Universität Berlin, Humboldt-Universität zu Berlin, and Berlin Institute of Health, Berlin, Germany.

*Correspondence: angela.relogio@medicalschool-hamburg.de; elena.braicu@charite.de

This PDF file includes:

Figure S1: Descriptive analysis of patient reported outcomes

Figure S2: Gene expression changes and circadian shifts among patients in response to rucaparib therapy

Figure S3: Spearman correlation heatmap (box-level) showing relationships between gene expression levels, circadian properties, and patient reported outcomes in the Circadian Cohort at baseline, placebo, rucaparib, and post-rucaparib treatment stages.

Figure S4: Spearman correlation heatmap (patient-level) displaying only significant correlation pairs between gene expression, circadian properties, and patient reported outcomes at baseline, placebo, rucaparib, and post-rucaparib treatment stages.

Figure S5: Spearman correlation heatmap (box-level) showing only significant correlation pairs between gene expression, circadian properties, and patient reported outcomes at baseline, placebo, rucaparib, and post-rucaparib treatment stages.

Figure S6: Patient reported quality of life (QoL) outcomes and adverse events (AE) in the MAMOC rucaparib cohort, illustrating QoL deterioration and an increase in AE across treatment stages compared to the placebo group.

Figure S7: Circadian dysregulation in the rucaparib group showing association with adverse events.

Figure S8: Circadian dysregulation in the placebo group showing no association with adverse events.

Figure S9: Circadian network changes and gene expression in response to rucaparib therapy vs. placebo.

Figure S10: Graphical representation of the interactions between core-clock genes and transporters/enzymes related to rucaparib drug activity.

Figure S11: Mathematical modelling used to predict drug toxicity over time.

Figure S12: Spearman correlation analysis reveals the connection between toxicity outcomes, quality of life, and adverse events.

Figure S13: Relationship between circadian gene expression and patient reported outcomes in response to rucaparib therapy compared to placebo.

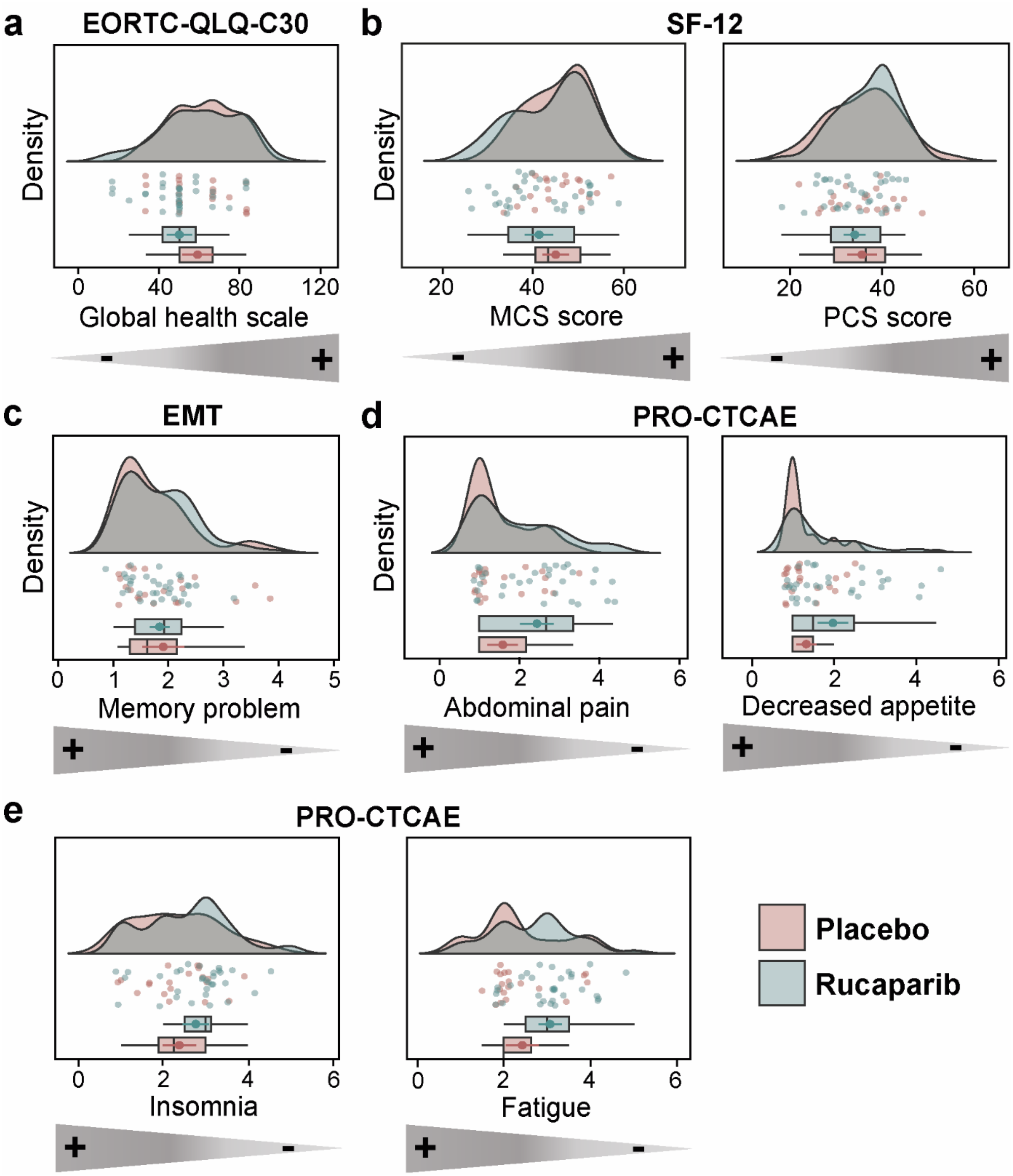

Figure S1: Descriptive analysis of patient reported outcomes. *Patients from both placebo and rucaparib groups were provided with a specific set of questionnaires during treatment to evaluate overall physical/mental health domain and adverse events. + represents less negative effects and – represents more negative effects. The density plot shows the distribution of the full MAMOC cohort (14 placebo, 28 rucaparib), while the jitter plots and boxplots represent the Circadian cohort (5 placebo, 10 rucaparib).* ***a.*** *The rucaparib group showed deteriorated global health scale vs. the placebo.* ***b.*** *SF-12 questionnaires showed altered physical and mental functioning in the rucaparib group compared to the placebo group (MCS: Mental Component Summary; PCS: Physical Component Summary).* ***c.*** *Memory problem showed no differences between rucaparib and placebo groups.* ***d-e.*** *PRO-CTCAE evaluated symptomatic toxicity. The rucaparib group showed an increased incidence of abdominal pain, decreased appetite, insomnia and fatigue than the placebo group (Statistical test used: Wilcoxon rank-sum test with BH correction; error bars are 95% confidence intervals).*

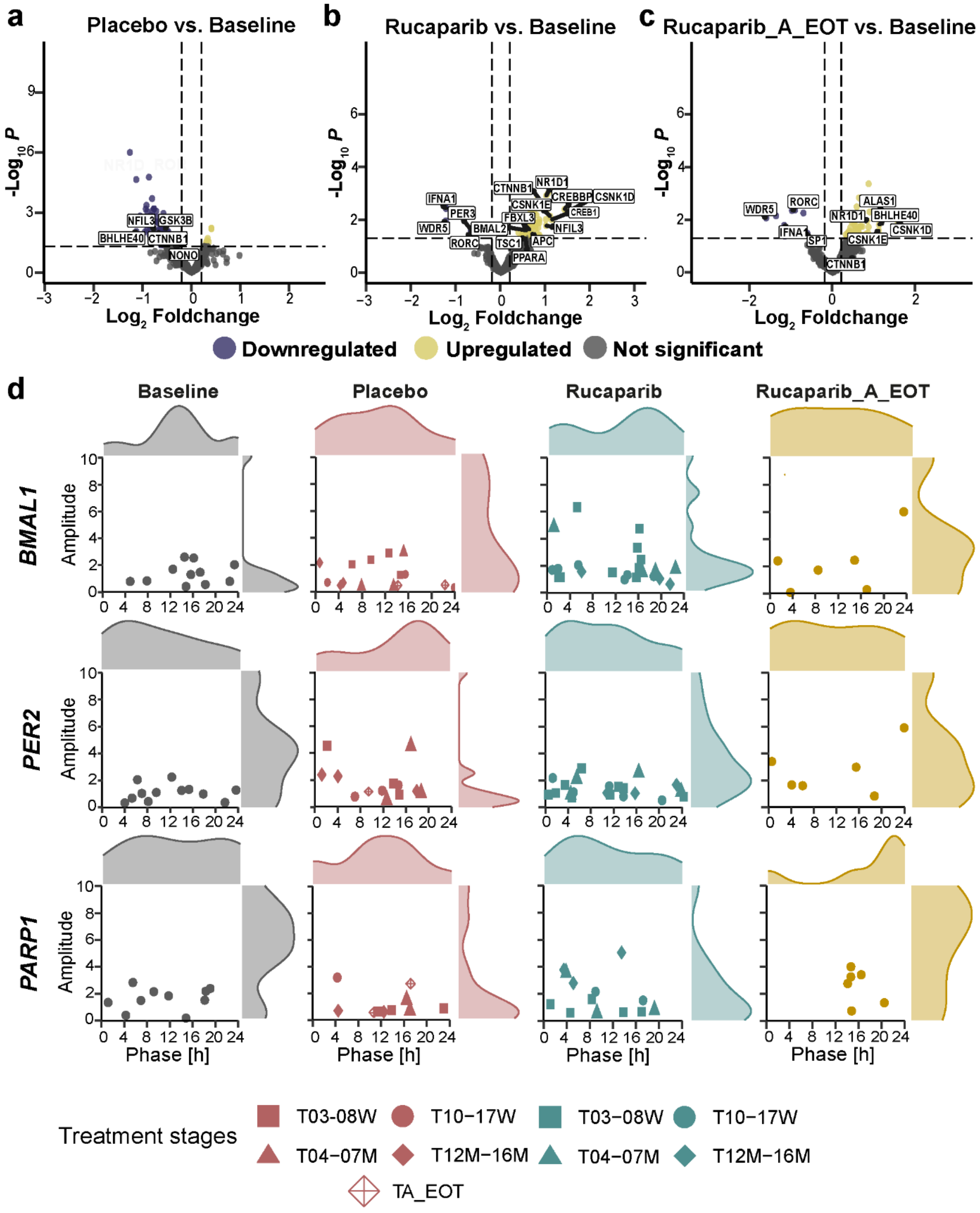

***Figure S2: Gene expression changes and circadian shifts among patients in response to rucaparib therapy. a-c**. Volcano plot of differentially expressed genes. The plot compares gene expression changes in: **a**. Placebo vs. Baseline, **b**. Rucaparib vs. Baseline, and **c.** Rucapatib_A_EOT vs. Baseline. The x-axis shows log2 fold change, and the y-axis represents -log10 p-value. Few of the significant upregulated or downregulated genes are highlighted, showcasing key expression differences between treatment groups. **d.** Acrophase density plot illustrates the changes in circadian properties (amplitude and phase) of BMAL1, PER2, and PARP1 genes due to rucaparib therapy or no therapy. Each dot represents an individual patient sampling box (8 samples over the course of two days) collected at different treatment stages, highlighting the variability in circadian phase shifts and amplitude changes across patients over time (A_EOT: after the end of treatment).*

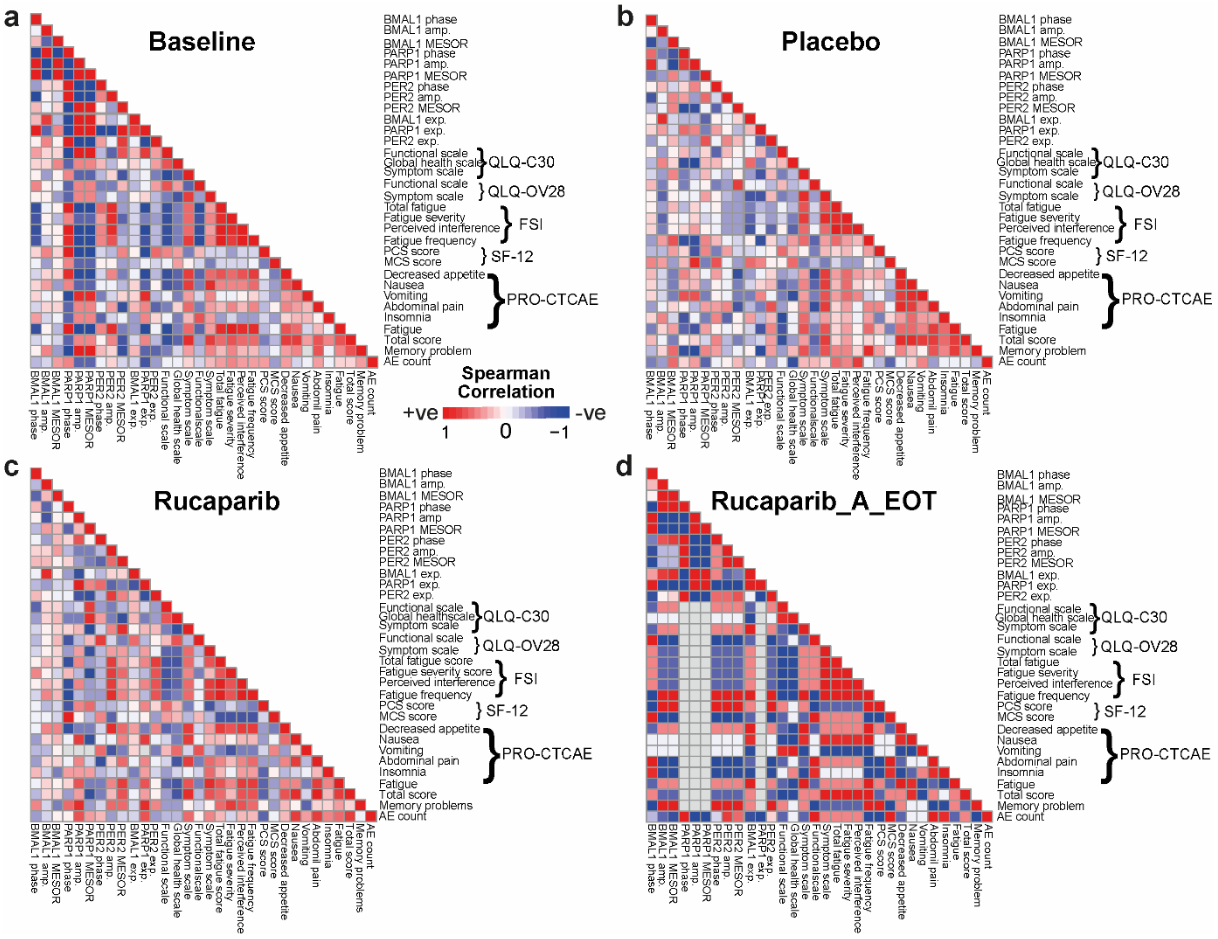

Figure S3: Spearman correlation heatmap (box-level) showing relationships between gene expression levels, circadian properties, and patient reported outcomes in the Circadian Cohort at baseline, placebo, rucaparib, and post-rucaparib treatment stages. *Since each patient completed questionnaires and provided saliva samples at multiple time points, the correlation analysis was performed at the individual sampling box-level (A_EOT: after the end of treatment).*

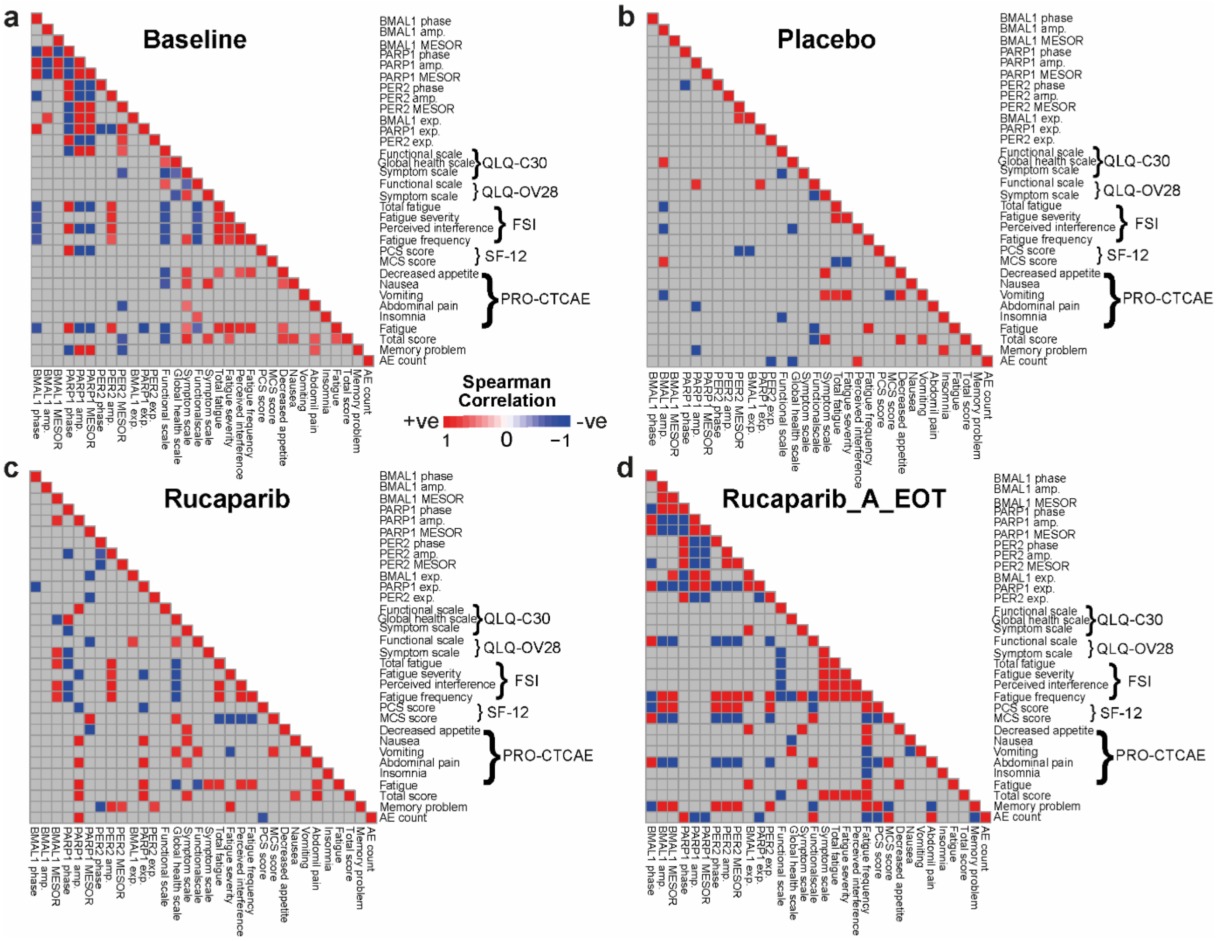

Figure S4: Spearman correlation heatmap (patient-level) displaying only significant correlation pairs between gene expression, circadian properties, and patient reported outcomes at baseline, placebo, rucaparib, and post-rucaparib treatment stages. *The correlation analysis was performed at the patient level (A_EOT: after the end of treatment).*

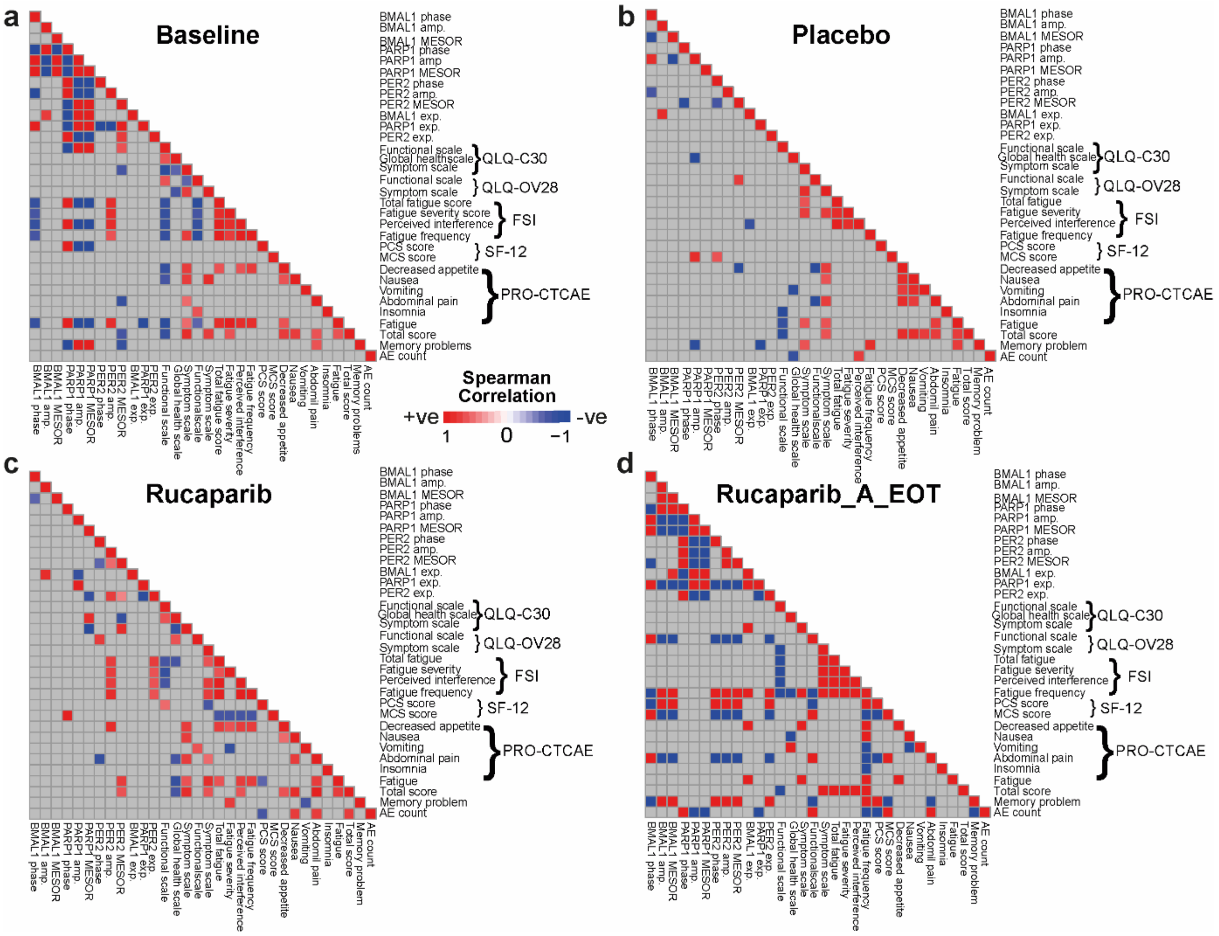

Figure S5: Spearman correlation heatmap (box-level) showing only significant correlation pairs between gene expression, circadian properties, and patient reported outcomes at baseline, placebo, rucaparib, and post-rucaparib treatment stages. *Since each patient completed questionnaires and provided saliva samples at multiple time points, the correlation analysis was performed at the individual sampling box-level (A_EOT: after the end of treatment).*

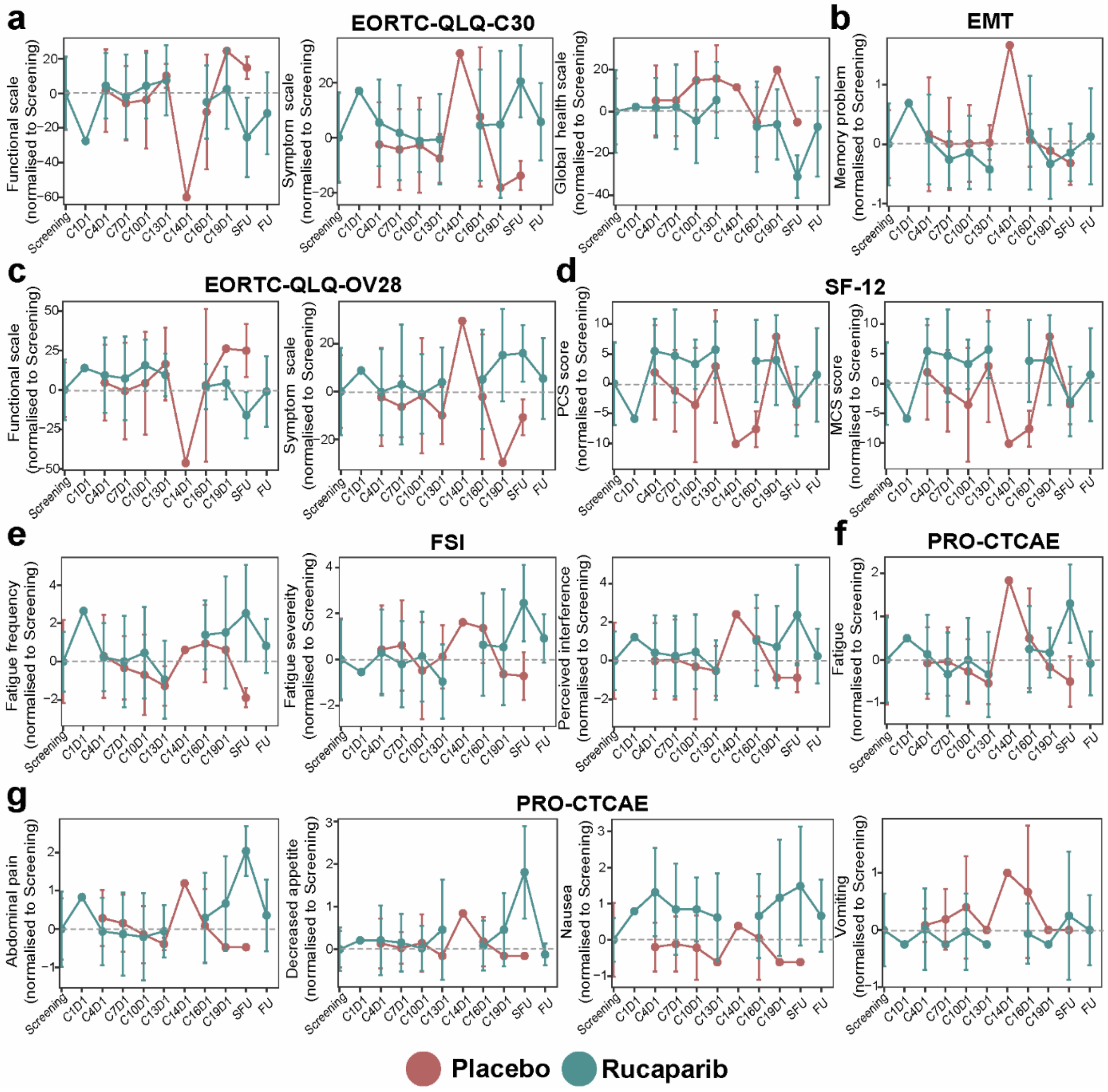

Figure S6: Patient reported quality of life (QoL) outcomes and adverse events (AE) in the MAMOC rucaparib cohort, illustrating QoL deterioration and an increase in AE across treatment stages compared to the placebo group. *Questionnaire results are visualised across various treatment timepoints (C: cycle; D: day; SFU: safety follow-up; FU: follow-up; only one patient completed the questionnaire during C1D1 and C14D1). Error bars are defined as Mean± SD.*

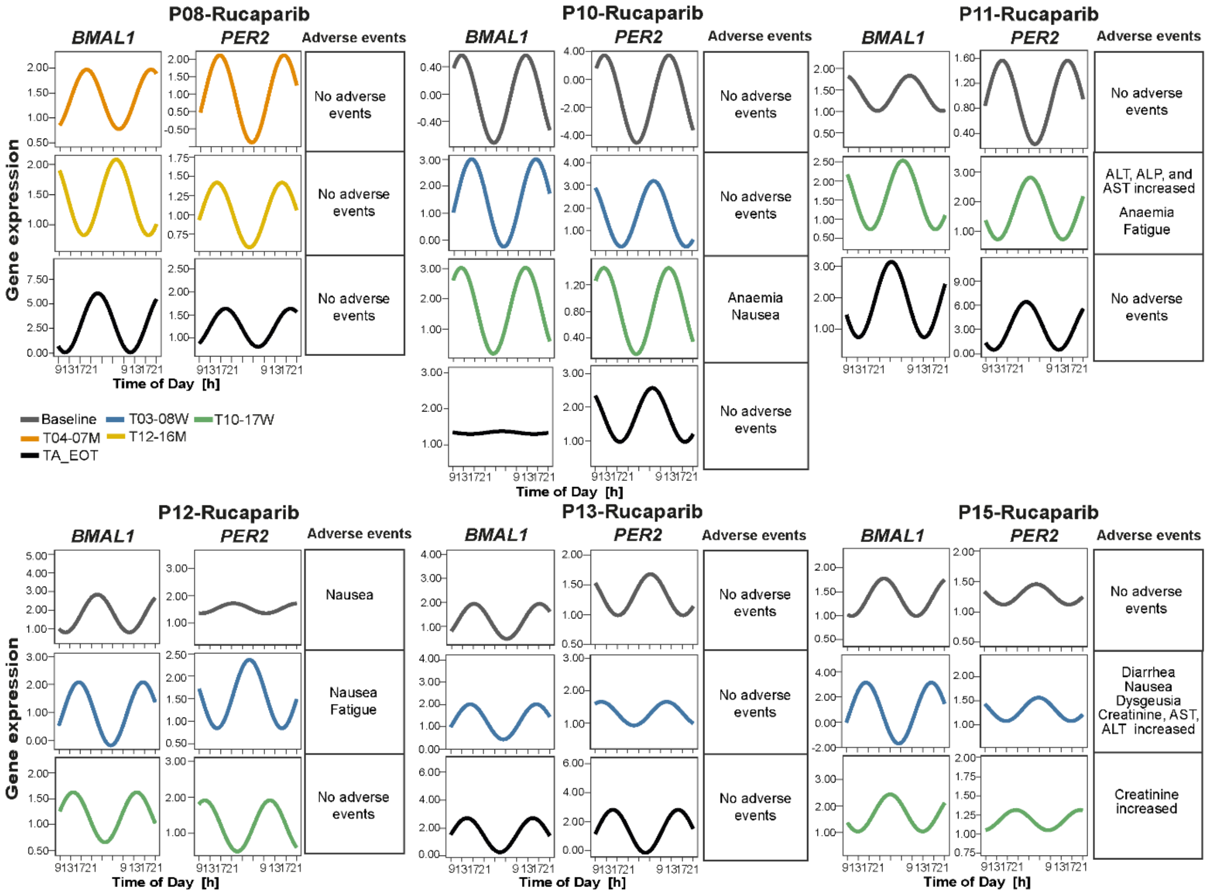

Figure S7: Circadian dysregulation in the rucaparib group showing association with adverse events. *Line graphs display the circadian expression patterns of core clock genes (BMAL1 and PER2) across the treatment period for each patient, illustrating individual variations in circadian rhythms over time and their association with reported adverse events (TA_EOT: time after the end of treatment).*

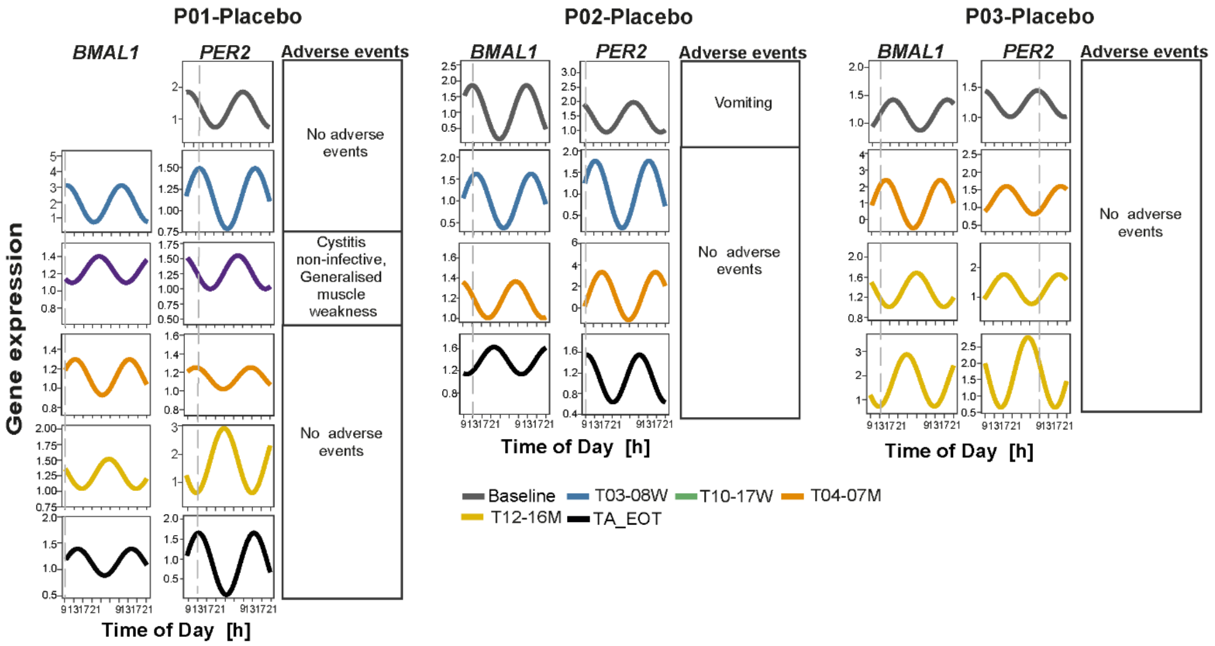

Figure S8: Circadian dysregulation in the placebo group showing no association with adverse events. *Line graphs display the circadian expression patterns of core clock genes (BMAL1 and PER2) across the treatment period for each patient, illustrating individual variations in circadian rhythms over time.*

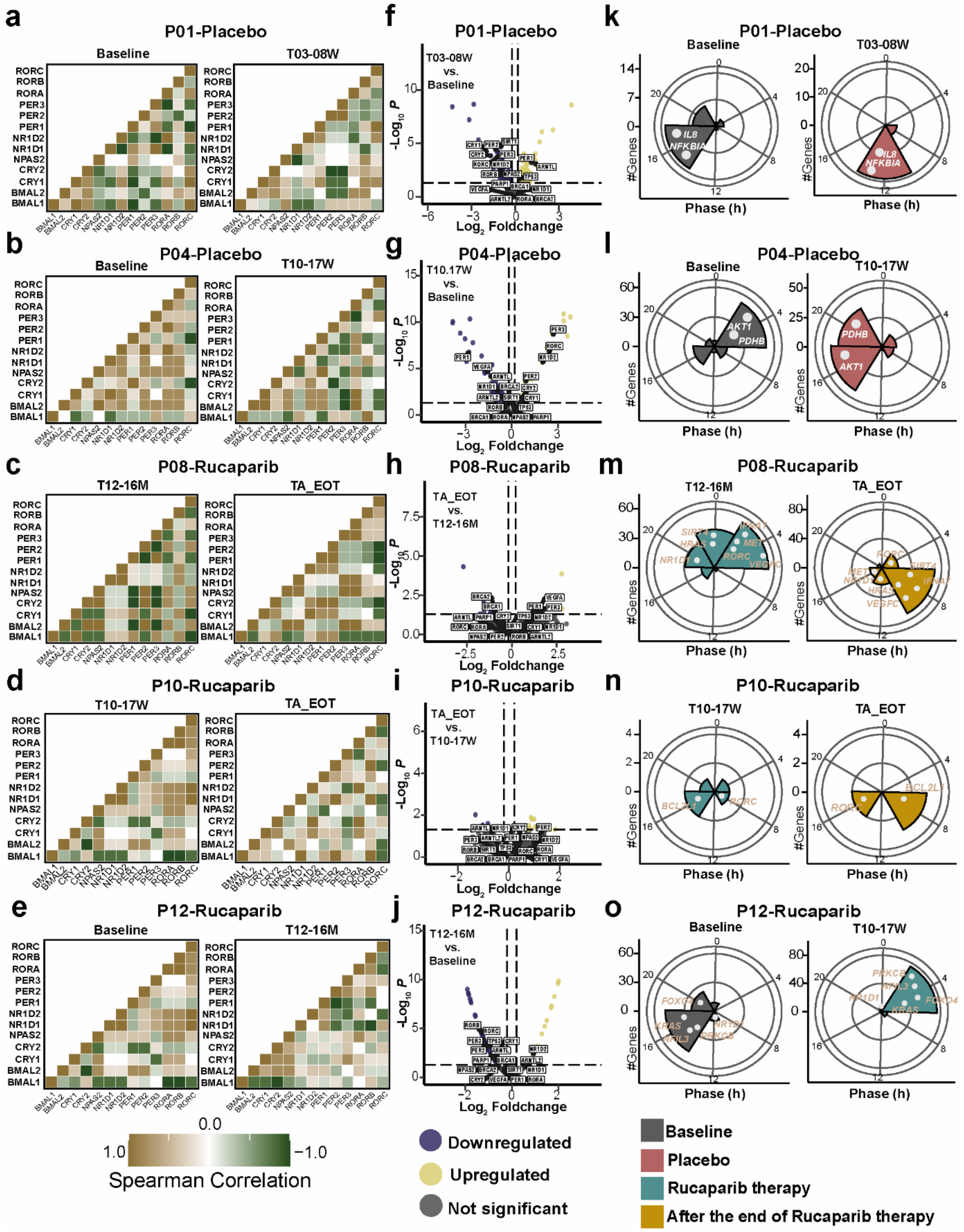

Figure S9: Circadian network changes and gene expression in response to rucaparib therapy vs. placebo. *To investigate alterations in circadian gene expression, its properties, and differential rhythmicity between the rucaparib and placebo groups, we analysed individual patient data.* ***a-e.*** *The heatmap illustrates variations in correlations between key core-clock genes, highlighting how treatment influences the connectivity and interactions within the circadian network.* ***f-j.*** *Volcano plots showing differential gene regulation among patients treated with rucaparib or placebo compared to their respective controls. Each point represents a gene, with the x-axis showing log2 fold change and the y-axis displaying -log10 p-value, highlighting significant upregulation and downregulation of gene expression in response to treatment.* ***k-o.*** *Acrophase bin plots representing the distribution of genes exhibiting differential rhythmicity (phase shifts) among rucaparib and placebo patients compared to their*

controls. More core-clock and cancer-associated genes were differentially rhythmic in the rucaparib-treated patients.

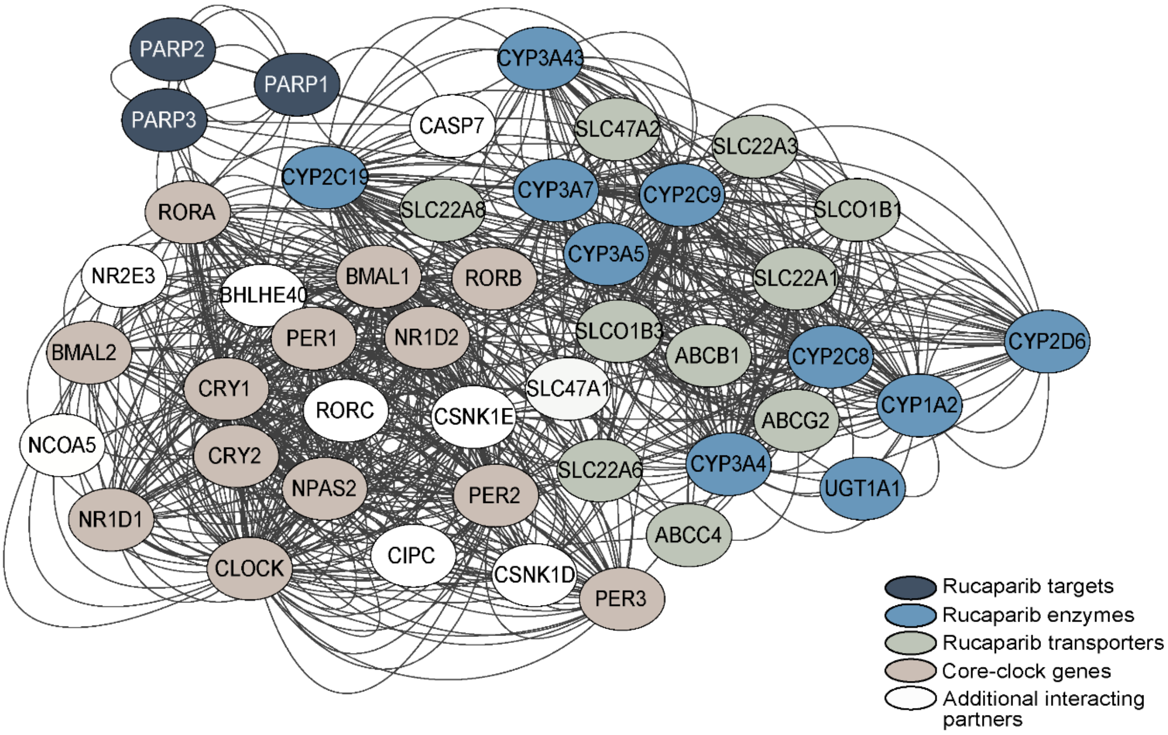

Figure S10: Graphical representation of the interactions between core-clock genes and transporters/enzymes related to rucaparib drug activity.

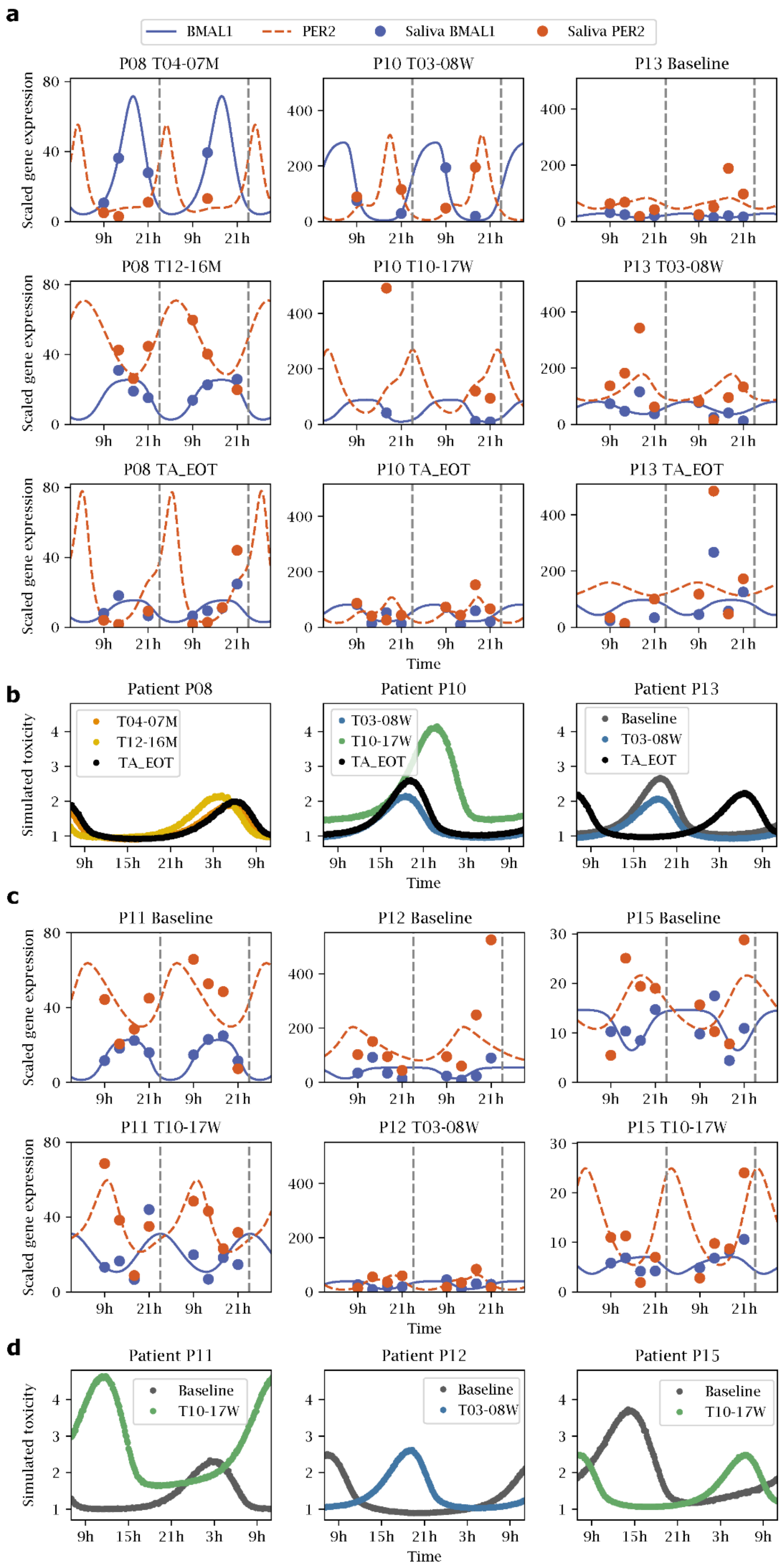

a
BMAL1
PER2
Saliva BMAL1
Saliva PER2
P08 T04-07M
P10 T03-08W
P13 Baseline
P08 T12-16M
P10 T10-17W
P13 T03-08W
P08 TA_EOT
P10 TA_EOT
P13 TA_EOT
Scaled gene expression
Time
b
Patient P08
Patient P10
Patient P13
T04-07M
T12-16M
TA_EOT
T03-08W
T10-17W
Baseline
Simulated toxicity
c
P11 Baseline
P12 Baseline
P15 Baseline
P11 T10-17W
P12 T03-08W
P15 T10-17W
d
Patient P11
Patient P12
Patient P15

Figure S11: Mathematical modelling used to predict drug toxicity over time. a. *Saliva gene expression (dots) and personalised network dynamics for PER2 and BMAL1 for patients P08, P10, and P13-Rucaparib. Note the small number of sampling times for P10-Rucaparib.* ***b.*** *Predicted toxicity curves given the personalised network.* ***c.*** *Saliva gene expression (dots) and personalised network dynamics for PER2 and BMAL1 for patients P11-Rucaparib, P12-Rucaparib, and P15-Rucaparib.* ***d.*** *Predicted toxicity curves given the personalised network.*

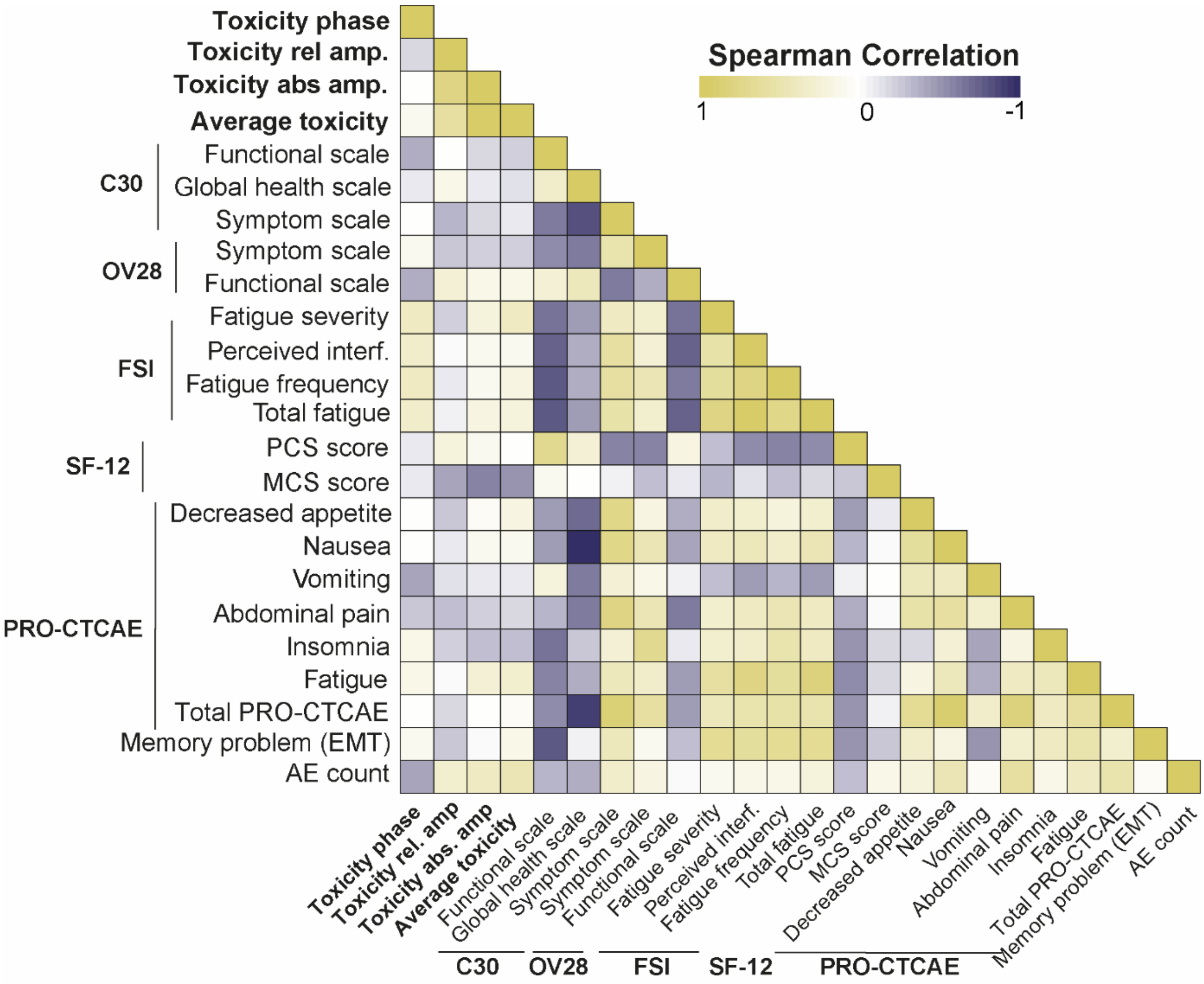

Figure S12: Spearman correlation analysis reveals the connection between toxicity outcomes, quality of life, and adverse events *(MCS: Mental Component Summary; PCS: Physical Component Summary)**.***

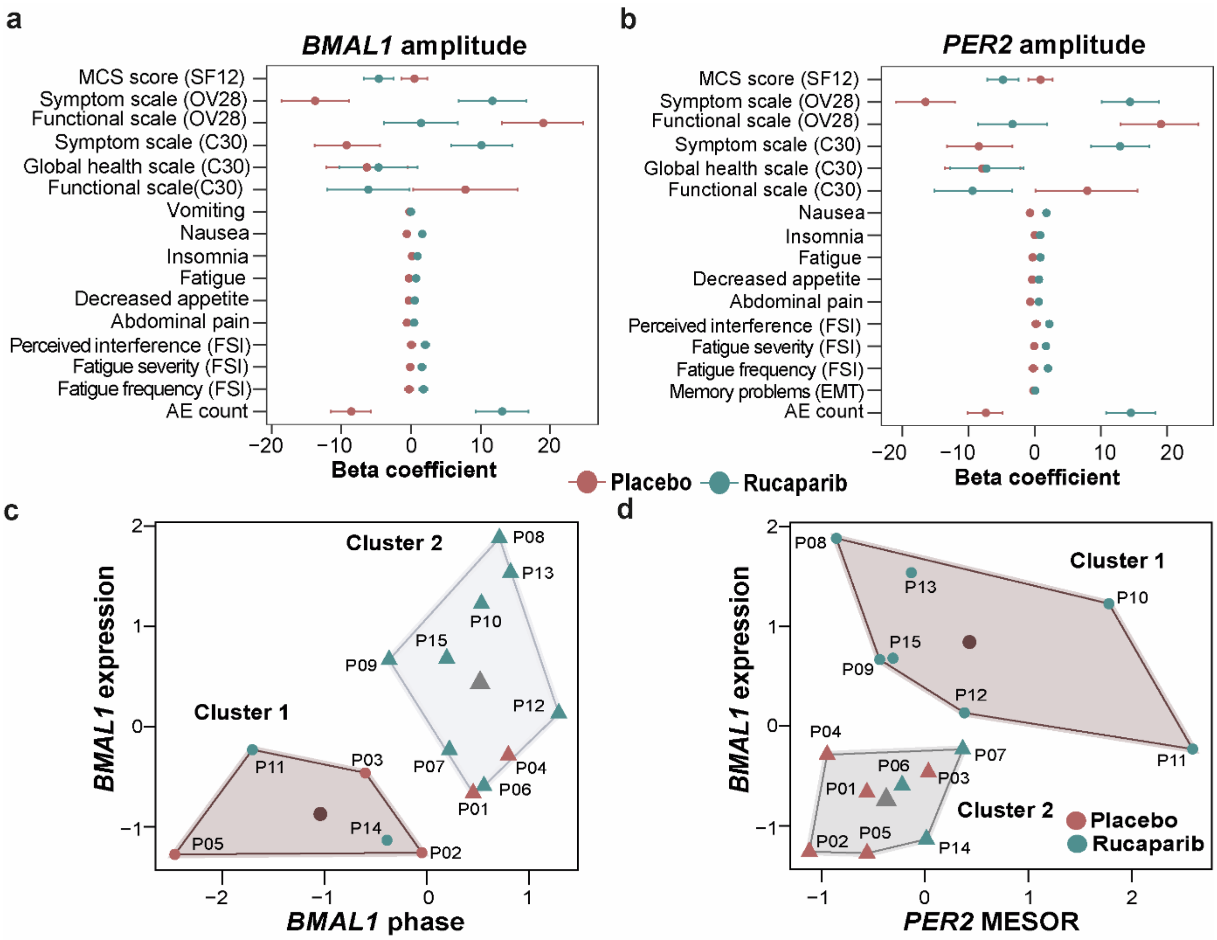

Figure S13: Relationship between circadian gene expression and patient reported outcomes in response to rucaparib therapy compared to placebo. a-b. *OLS with bootstrapping analysis illustrates the relationships between the amplitude of BMAL1 and PER2 genes and various patient reported outcomes. This analysis highlights how fluctuations in the amplitude of these core clock genes correlate with patient health indicators, suggesting potential implications for treatment responses and circadian rhythm effects on overall well-being. The error bar represents 95% CI (MCS: Mental Component Summary; PCS: Physical Component Summary).* ***c-d.*** *K-means clustering analysis identifies two distinct clusters based on circadian features of BMAL1 and PER2, showing differences in patient groupings between the placebo and rucaparib groups. This clustering suggests varying circadian characteristics among patients, with implications for treatment responses and individualised care strategies.*
